# Gradient boosting-based discrete failure time model for selecting time-varying effects and interactions

**DOI:** 10.1007/s10985-026-09710-4

**Published:** 2026-05-13

**Authors:** Lingfeng Luo, Kevin He, Jeremy M. G. Taylor

**Affiliations:** Department of Biostatistics, University of Michigan, Ann Arbor, MI USA

**Keywords:** Discrete failure time, High dimensional variable selection, Hierarchical interactions, Survival analysis, Time-varying effects

## Abstract

Analyzing survival data from the National Cancer Institute’s Surveillance, Epidemiology, and End Results (SEER) Program offers invaluable insight to guide cancer management. In these data the event times are recorded in units of months. In such datasets, it can be important to consider the selection of covariates, distinguish between time-varying and time-independent effects, and capture important interaction terms. However, variable selection is difficult to combine with the differentiation between time-varying and time-independent effects. Moreover, accurately selecting interaction terms is more challenging because of the hierarchy restriction that an interaction term should only enter a model if one or both associated main effects are also included. To resolve these issues, we develop a coordinate ascent-based gradient boosting procedure for discrete failure time models. This approach achieves variable selection in high-dimensional settings, differentiates between time-varying and time-independent variables, and selects important interaction terms under hierarchy restrictions. Traditional boosting stopping criteria are ad hoc, while preferable information-criteria-based rules necessitate defining degrees of freedom, a challenge in survival models. Our approach provides well-defined degrees of freedom and offers information-criteria-based stopping rules. Simulation studies show the proposed method achieves good selection performance. We apply the proposed method to SEER melanoma cancer data and select important risk factors, distinguish between time-varying and time-dependent effects and detect important two-way interactions.

**Supplementary Information:**

The online version contains supplementary material available at 10.1007/s10985-026-09710-4.

## Introduction

Discrete failure time models provide a powerful tool in large-scale registries where event times are recorded on a discrete scale (e.g., months). These models allow for a direct estimation of the baseline hazard and the computation of survival probabilities at specific time intervals. In contrast to continuous-time approaches, discrete models naturally accommodate the large number of ties in event times that frequently arise in registry-based survival studies. However, classical discrete failure time models often assume proportional hazards and linear additive effects of covariates. With modern large-scale datasets, it is possible to consider more flexible models. The particular flexibility we consider in this paper is to allow for two-way interactions between covariates and to allow for time-varying effects of covariates. This degree of flexibility is considerable and in many applications will be sufficient to adequately describe the data, while retaining the ability to interpret the estimated parameters. This added flexibility is achieved by adding parameters to the model, to represent both the interactions and the time-varying effects. Another situation is high dimensional data when there is a very large number of covariates. Even if interactions were not considered, there would still be a large number of parameters for models in this situation. Models with high numbers of parameters introduce some challenges, both computational and statistical. One approach to address these problems is to set many parameters to have zero values, which corresponds to variable selection in the high dimensional covariate setting or removing interactions in our flexible model. Setting parameter values to zero is also appealing as it makes the model more parsimonious, which aids interpretation. In summary, when there are many covariates, it is challenging to select those that have significant effects, and distinguish between time-varying and time-dependent effects. Similarly, it can be challenging to select the important interactions from the large number of choices, while allowing for time-varying effects, even with a modest number of covariates.

In many medical studies interaction terms can be especially important, where the effects of personal health behaviors, as well as genetic, environmental, and demographic factors, can depend on each other and vary over time. Several studies have shown the value of identifying interaction terms in survival data. For instance, Kim et al. (2019) proposed a method for detecting gene-gene interactions, while Schwender and Ickstadt (2008) discovered an interaction between tobacco and alcohol risk factors and racial groups in esophageal squamous cell carcinoma. Another example is the study by Ferris et al. (2016), which highlighted the importance of considering interactions between the year of diagnosis and cancer stage, as they found improved survival outcomes for patients with recurrent or metastatic squamous cell carcinoma of the head and neck treated with Nivolumab in recent years.

Given these findings, it is clear that identifying and incorporating interaction terms represent a crucial aspect of statistical modeling in the study of complex diseases or data from clinical trials. The ability to account for these interactions not only reveals the underlying mechanisms driving disease outcomes, but also sheds light on the inherent heterogeneity of these outcomes. Therefore, refining the process for selecting interaction terms is essential for advancing our understanding of disease progression and treatment efficacy.

While interaction terms play a crucial role in understanding the complex relationships between covariates, it is equally important to recognize the varying influence of covariates over time. For instance, tumor aggressiveness’ impact on survival rates for breast cancer patients receiving neoadjuvant chemotherapy diminishes after five years, while the risk persists for patients with positive hormone receptor status (Baulies et al. 2015). A recent study (Syn et al. 2021) highlights the importance of considering the changing impact of prognostic factors in oncology studies with long follow-ups. In particular, for colon cancer, they discovered that the negative impact of moderate-to-high tumor grade diminished after 26 months. For the first 10 months after surgery, right-sided tumors had a worse prognosis compared to left-sided tumors. However, after 129 months, right-sided tumors provided a survival benefit, resulting in a higher long-term survival rate compared to left-sided tumors. Another recent paper (Abrahamowicz et al. 2025) advocated for the use of time-varying coefficient hazard models and gave examples of their interpretation. These observations necessitate an efficient model selection procedure capable of differentiating between three types of covariates: (1) those excluded from the model, (2) those included with time-independent coefficients, and (3) those included with time-varying coefficients.

This research was motivated by studying the risk-factors for mortality in different types of cancers using data from the National Cancer Institute Surveillance, Epidemiology, and End Results (SEER) Program. Mortality rates among cancer patients vary greatly, which merits special scrutiny of the associated risk factors. Baseline covariates obtained from the SEER Program are frequently used for risk adjustment, and the Cox proportional hazards model has been commonly used. Analyzing the large-scale survival data from the SEER Program may Help guide the management of cancer. Detecting and characterizing the time-varying effects of factors collected at the time of diagnosis could reveal important and useful patterns. In a recent paper Roberts et al.( 2024) considered data from 14 different cancer sites and showed that the covariates age and stage of disease had time-varying coefficients for most of the cancer sites. While these authors considered time-varying effects in a continuous time model, using the methodology described in Luo et al.( 2023), they did not consider interactions. In this paper we illustrate the approach we develop in the setting of survival after metastatic melanoma, based on a dataset of over 10,000 patients from the SEER database. In the last 15 years immunotherapy has been increasingly used to treat metastatic melanoma based on studies that have shown substantially improved survival compared to previously used treatments. A question of interest is whether the improvement is larger in some patient subgroups compared to others. This is naturally captured by including interactions in the model.

In large-scale disease registries, such as the SEER Program, event times are frequently recorded on a discrete scale (e.g. months), and ties in event times can be numerous, posing a challenge for the application of continuous-time models. To address this issue, the use of a discrete survival model is indicated (Kalbfleisch and Prentice 2002; Tutz and Schmid 2016). These models allow for a direct estimation of the baseline hazard and facilitate the computation of survival probabilities at specific time intervals, such as monthly survival probabilities for cancer patients. Moreover, employing discrete survival models helps avoid biases associated with continuous-time models and accounts for the differences in time scales for different event types, ultimately leading to more accurate and reliable estimation and prediction (Lee et al. 2018, 2019).

### Related work

A variety of variable selection techniques have been developed for traditional survival analysis with varying coefficients. Lin and Zhang (2006) proposed a component selection and smoothing operator using an L1 norm penalty, which was later extended to varying coefficient Cox models by Leng and Zhang (2006). Yan and Huang (2012) and Möst and Pößnecker (2016) considered Group-LASSO-based methods that select important time-varying coefficients. However, the simultaneous selection of time-varying and fixed coefficients, inclusion or exclusion of covariates, and potential interaction terms remains largely unexplored in survival analysis. For instance, selecting relevant interaction terms poses a unique challenge, especially under hierarchical constraints. The strong hierarchy constraint requires that an interaction term be included only if both corresponding main effects are included, whereas the weak hierarchy only requires one of the main effects to be present. While the LASSO (Tibshirani 1997) is a widely used technique for variable selection, it has limitations when modeling interactions, as standard LASSO does not guarantee adherence to hierarchical constraints. Choi et al. (2010) extended the LASSO to simultaneously fit regression models and identify important interaction terms. Similarly, methods developed by Choi et al. (2010) and Bien et al. (2013) address hierarchical constraints in interaction selection. However, these methods lack the flexibility to differentiate between time-varying and time-invariant effects, limiting their applicability in survival settings where temporal dynamics are of interest.

Our proposed solution is motivated by boosting, originally introduced by Freund (1996) for binary classification. Friedman et al. (2000) and Friedman (2001) extended boosting to handle various loss functions and laid out a gradient boosting framework. For high-dimensional variable selection, Bühlmann and Yu (2003, 2006) proposed $\ell _2$-Boosting procedure and demonstrated the effectiveness of its component-wise procedure. This approach can be viewed as a type of forward selection. To achieve variable selection in high-dimensional settings, He et al. (2015) proposed a modified gradient boosting for Cox proportional hazards models based on the Minorization-Maximization algorithm (Lange 2012), and He et al. (2021) further extended the boosting procedure for Cox models with time-varying effects. More recently, De Bin and Stikbakke (2023) developed a boosting algorithm for first-hitting-time models in high-dimensional frameworks, and demonstrated improved prediction accuracy through the integration of clinical and molecular data.

Gradient boosting-based methods have also been proposed in the context of discrete failure time models. Utilizing the fact that the likelihood of a discrete survival model is equivalent to that of a generalized linear model of longitudinal binary outcomes, Tutz and Schmid (2016) and Berger et al. (2023) discussed the extension of classical gradient boosting to discrete failure time models based on augmented discrete time-to-event data. Tree-based boosting methods have also been proposed for discrete failure time models (Bou-hamad et al. 2009, 2011; Schmid et al. 2014). These methods rely on an expansion of the original dataset in a long format, where at each discrete time point, the covariate values and event status for at-risk subjects are stacked to create a massive augmented discrete time-to-event dataset. Each observation in the augmented discrete time-to-event data corresponds to a longitudinal binary outcome, i.e. the event status for each patient at each time point for which the patient is still at risk. With the augmented data, classical gradient boosting procedures (e.g. $\ell _2$-Boosting) for binary outcomes can then be applied. However, the computational cost increases quickly as the sample size or the number of predictors grows, which easily overwhelms powerful computers. Thus, these expansion-based approaches suffer from serious computational limitations, limiting their application to large-scale studies.

Another challenge for boosting methods is determining the optimal stopping iteration, which is critical for preventing overfitting and ensuring accurate predictions. Traditional approaches often rely on arbitrarily chosen stopping points, which can lead to computational inefficiency and increase the risk of overfitting or underfitting. To address this, Mayr et al. (2012) proposed a data-driven stopping rule for boosting algorithms that combines resampling methods with AIC-based information criteria. Bühlmann and Hothorn (2007) and Hofner et al. (2011) introduced the concept of degrees of freedom within the generalized linear model framework and defined an AIC criterion accordingly. Berger et al. (2023) extended this concept to discrete failure time models; however, the associated degrees of freedom are computed using augmented data, which leads to the aforementioned computational challenges.

### Our contribution

To address these challenges, we propose a novel boosting procedure for discrete failure time models. Compared with existing methods, our proposed approach offers several distinct advantages. First, it introduces a flexible model framework that effectively differentiates between time-varying, time-independent, and non-significant variables. Second, it effectively identifies crucial interaction terms, following both strong and weak hierarchical constraints, a fundamental aspect for understanding complex relationships in survival data. Third, the boosting procedure does not require the expansion of the dataset into a long format, making it feasible for studies with large sample sizes. Fourth, we introduce computationally efficient information criteria-based stopping rules, enhancing the model’s precision and preventing overfitting.

The remainder of this article is organized as follows: In Sect. 2, we first provide notation and then introduce the proposed gradient boosting-based algorithm for discrete failure time models. The performance of the proposed method is examined using simulations in Sect. 3 and applications to the analysis of SEER data in Sect. 4. The article concludes with a brief discussion in Sect. 5.

## Method

### Discrete failure time models with time-varying effects

Let $D_i$ denote the event time of interest and $C_i$ be the censoring time for subject *i*, $i=1,\ldots , n$, where *n* is the sample size. The observed event time is $T_i=\min \{D_i,C_i\}$. Let $t_1, \ldots , t_S$ be the distinct failure times indexed by $s=1, \ldots , S$ and $t_0 = 0$. Let $\mathcal {D}_{s}$ and $\mathcal {C}_{s}$ denote the individuals failing and censored at time $t_s$, respectively. Let ${\textbf {Z}}_{i}$ be a P-dimensional covariate vector for the *i*-th subject, $i=1,\ldots ,n$. These covariates are measured at time zero and have no missing values. Assume that $D_i$ and $C_i$ are independent, given the value of ${\textbf {Z}}_{i}$. Let $\lambda (t_s;{\textbf {Z}}_{i})=P(D_i=t_s|D_i\ge t_s, {\textbf {Z}}_{i})$ be the hazard function at time $t_s$ for the *i*-th subject with covariate ${\textbf {Z}}_{i}$. The likelihood function is given by

1 $$\begin{aligned} L=\prod _{s=1}^{S}\left\{ \prod _{i \in \mathcal {D}_{s}} h(t_s;{\textbf {Z}}_{i}) \prod _{i \in \mathcal {C}_s} S(t_s;{\textbf {Z}}_{i})\right\} , \end{aligned}$$

where $S(t_s;{\textbf {Z}}_{i})=P(D_i>t_s|{\textbf {Z}}_{i})= \prod _{\ell =1}^s \{1-\lambda (t_{\ell };{\textbf {Z}}_{i})\}$ is the survival function and $h(t_s;{\textbf {Z}}_{i})=P(D_i=t_s|{\textbf {Z}}_{i})= \lambda (t_{s};{\textbf {Z}}_{i})\prod _{\ell =1}^{s-1} \{1-\lambda (t_{\ell };{\textbf {Z}}_{i})\}$ is the mass function of subject *i* at time $t_s$.

To estimate the non-proportional hazards model allowing for interaction terms, we consider a general formulation of the hazard

$$\begin{aligned} \lambda (t_s;{\textbf {Z}}_{i}) = g\left\{ \gamma _s + f(t_s; \textbf{Z}_i)\right\} , \end{aligned}$$

where $\gamma _s$ is the link function transformed baseline hazard at time *s*, *g* is the inverse of the link function, and

$$\begin{aligned} f(t_s; \textbf{Z}_i) =&\sum _{j=1}^P\phi _jz_{ij} + \sum _{j=1}^P\beta _{j}(t_s)z_{ij} + \sum _{j< j^{'}}\alpha _{jj^{'}}z_{ij}z_{ij^{'}},\end{aligned}$$

where $\phi _j$ is the time-independent effect of the *j*th covariate, $\beta _j(t)$ is the time-varying effect of the *j*th covariate, and $\boldsymbol{\alpha } = (\alpha _{jj^{'};\ 1\le j < j^{'} \le P})^\top$ are the coefficients of the interaction terms.

To specify the time-varying coefficients for $\boldsymbol{\beta }(t) = (\beta _1(t), \beta _2(t), \ldots , \beta _P(t))$, we use B-splines with a particular parameterization. We define

$$\begin{aligned} \beta _j(t) = \sum _{k=2}^K\theta _{jk}B_k(t), \ j = 1,\ldots ,P, \end{aligned}$$

where $\textbf{B}^*(t) = \{B_1(t), \dots , B_K(t)\}^\top$ forms the usual B-spline basis, and the reduced basis function $\textbf{B}(t)$ is defined as

$$\begin{aligned} \textbf{B}(t)&= \left\{ B_2(t), \dots , B_K(t)\right\} ^\top \subset \textbf{B}^*(t). \end{aligned}$$

*K* is the number of basis functions and $\boldsymbol{\theta }_j = (\theta _{j2},\ldots ,\theta _{jK})$ is a vector of coefficients with $\theta _{jk}$ being the coefficient for the *k*th basis function of the *j*th covariate. We use $K=7$ throughout this paper. Restricting to a time-independent component is handled in this parameterization by requiring $(\theta _{j2},\ldots ,\theta _{jK}) = (0,\ldots ,0)$. To implement this specification, we employ function *bs* from *splines* package by setting intercept = FALSE. Therefore, we have

$$\begin{aligned} f(t_s; \textbf{Z}_i) = \sum _{j=1}^P\phi _jz_{ij} + \sum _{j=1}^P\boldsymbol{\theta }_j^\top \textbf{B}(t_s)z_{ij} + \sum _{j < j^{'}}\alpha _{jj^{'}}z_{ij}z_{ij^{'}}. \end{aligned}$$

We consider the logit link function (Kalbfleisch and Prentice 2002) in this paper, which leads to

2 $$\begin{aligned} \lambda (t_s;{\textbf {Z}}_{i}) = \frac{\exp (\gamma _s + f(t_s; \textbf{Z}_i))}{1+\exp (\gamma _s + f(t_s; \textbf{Z}_i))}. \end{aligned}$$

Thus, $\phi _j$, $\beta _j(t)$ and $\alpha _{j,j'}$ can be interpreted as log odds ratios.

### Overview of the procedure

To perform variable selection, distinguish between time-varying and time-independent effects, and detect important two-way interactions in high-dimensional settings, we propose a boosting procedure. We first introduce the challenges brought by interaction hierarchical conditions and time-varying hierarchical conditions. Then we give an overview of how the proposed procedure works.

In statistical modeling, the principle of strong hierarchy dictates that an interaction term can be included in a model only if both of its corresponding main effect terms are also included. Mathematically, to include the interaction term $z_jz_{j^{\prime }}$ (where $1\le j<j^{\prime }\le P$), the following holds:

$$\begin{aligned} \alpha _{jj^{\prime }} \ne 0 \Longrightarrow \left\{ \phi _j \ne 0 \text { or } \boldsymbol{\theta }_j \ne 0\right\} \text { and } \left\{ \phi _{j^{\prime }} \ne 0 \text { or } \boldsymbol{\theta }_{j^{\prime }} \ne 0\right\} . \end{aligned}$$

We only consider strong hierarchical structure in this section. Extension to weak hierarchical structure or no hierarchy structure is straightforward.

Our proposed procedure employs a forward selection approach. In each iteration, we select a single term from the following categories: time-independent effects, time-varying effects, and second-order interaction terms. The parameter estimates are then updated for the parameters already selected and for the parameters associated with the newly selected term. We note that the set of candidate terms, from which to select a single term, evolves dynamically. Initially, the candidate set contains only main effects. As the algorithm iterates, the procedure can add interaction terms to the candidate set, provided their corresponding main effects are already included in the model.

In addition to addressing standard hierarchy constraints for interactions, our proposed procedure is also flexible enough to account for time-varying hierarchy. This means a variable can be considered as having a time-varying effect only if it has been selected as a time-independent effect:

$$\begin{aligned} \boldsymbol{\theta }_j \ne 0 \Longrightarrow \phi _j \ne 0. \end{aligned}$$

We present the simulation results for time-varying hierarchy in the Online Appendix.

To formalize our algorithm, we introduce the following notation. For a non-censored observation ($$\delta _i = 1$$), define the sequence $$(y_{i1}, \ldots , y_{iT_i}) = (0, \ldots , 0, 1)$$, and for a censored observation ($$\delta _i = 0$$), define the sequence $$(y_{i1}, \ldots , y_{iT_i}) = (0, \ldots , 0),$$ where each $$y_{is}$$ encodes a transition from interval $$[t_{s-1}, t_s)$$ to $$[t_s, t_{s+1})$$ in the form:

$$\begin{aligned} y_{is}&= 1, \text {if individual i fails in}\ [t_{s-1},t_s) \\ y_{is}&= 0, \text {if individual i survives in}\ [t_{s-1},t_s). \end{aligned}$$

Let $\eta _{is} = \gamma _s + f(t_s; \boldsymbol{Z}_i)$, and define the vector $\boldsymbol{\eta }= (\eta _{11},\ldots ,\eta _{1T_1},\eta _{21},\ldots ,\eta _{nT_n})$, which is a vector of dimensional $\mathcal {N} = \sum _{i=1}^{n}T_i$. The corresponding log-likelihood to be maximized is given by:

3 $$\begin{aligned} \ell (\boldsymbol{\eta })&= \sum _{i=1}^n\sum _{s=1}^{T_i} \left[ y_{is}\eta _{is} -\log \left( 1+\exp \left( \eta _{is}\right) \right) \right] . \end{aligned}$$

Taking the first-order derivative with respect to $\boldsymbol{\eta }$, we have

4 $$\begin{aligned} \textbf{U}= \frac{\partial \ell (\boldsymbol{\eta })}{\partial \boldsymbol{\eta }}&= \left( y_{11} -\frac{\exp (\eta _{11})}{1+\exp (\eta _{11})}, \ldots , y_{nT_n} -\frac{\exp (\eta _{nT_n})}{1+\exp (\eta _{nT_n})} \right) , \end{aligned}$$

where $$U_{is} = y_{is} -\frac{\exp (\eta _{is})}{1+\exp (\eta _{is})}, i = 1,\ldots ,n, s=1,\ldots , T_i$$.

We define three index sets to represent the parameters corresponding to time-independent effects, time-varying effects, and interaction terms, respectively:

- Time-independent index set: $\textsf{P}_{\text {TI}} = \{1,\ldots ,P\}$, denoting the set of $\phi$ parameters.
- Time-varying index set: $\textsf{P}_{\text {TV}} = \{P+1,\ldots ,2P\}$, denoting the set of $\boldsymbol{\theta }$ parameters.
- Second-order interaction term index set: $\textsf{P}_{\text {INT}} = \{(j,j^{'}),1\le j < j^{'} \le P\}$, denoting the set of $\boldsymbol{\alpha }$ parameters.

We further define the candidate set – terms eligible for selection at iteration *m* of the boosting procedure – as a dynamically updated subset of the eligible index set. Under the weak or strong hierarchy conditions, the index set of interaction terms $\textsf{P}_{\text {INT}}$ is not directly used for parameter selection. Instead, the candidate set of the interaction term effects is dynamic across the iterations. To address this, we introduce additional notation to define our dynamic candidate set. For iteration step *m* of the boosting procedure, we define the following sets:

- $\textsf{G}_1^{[m]} = \{j: \hat{\phi }_j^{[m-1]} \ne 0\ \text {or}\ \hat{\boldsymbol{\theta }}_{j}^{[m-1]} \ne {\textbf {0}}, j = 1,\ldots ,P \}$: Non-zero time-independent or time-varying terms.
- $\textsf{G}_2^{[m]} = \{(j,j^{'}):\ j,j^{'}\in \textsf{G}_1^{[m]}, 1\le j<j^{'}\le P \}$: Potential non-zero interaction terms.

Hence, the candidate set for the iteration step *m* is $\textsf{P}_{\text {TI}}\cup \textsf{P}_{\text {TV}}\cup \textsf{G}_2^{[m]}$.

### Proposed DiscBoosting procedure

With the above notation in place, we propose the following iterative boosting procedure:

(a) Initialization: Consider a small positive value (e.g. 0.5) as the step size $\nu$. Initialize $\boldsymbol{\gamma }^{[0]} = \boldsymbol{0}, \boldsymbol{\phi }^{[0]} = \boldsymbol{0}, \boldsymbol{\theta }^ {[0]} = \boldsymbol{0}$ and $\boldsymbol{\alpha }^{[0]} = \boldsymbol{0}$. Then we let $\textbf{U}^{[0]} = \textbf{U}(\boldsymbol{\gamma }^{[0]}, \boldsymbol{\phi }^{[0]}, \boldsymbol{\theta }^ {[0]}, \boldsymbol{\alpha }^{[0]})$ based on equation 4. Also, we initialize the parameter candidate sets as $\textsf{P}_{\text {TI}}\cup \textsf{P}_{\text {TV}}\cup \textsf{G}_2^{[0]}$. Since $G^{[0]}_{1} = \emptyset$, we have $G^{[0]}_{2} = \emptyset$.

(b) Update $\textbf{U}$*:* Increase *m* by one: $m \leftarrow m + 1$. Compute the gradient $\textbf{U}= \frac{\partial \ell (\boldsymbol{\eta })}{\partial {\boldsymbol{\eta }}}$ and evaluate at $\boldsymbol{\eta }^{[m-1]}$:

$$\begin{aligned} U_{is}^{[m-1]} = \frac{\partial \ell (\boldsymbol{\eta })}{\partial {\eta _{is}}}\big |_{\eta _{is} = \eta _{is}^{[m-1]}},\ i=1,\ldots ,n,\ s=1,\ldots ,T_i. \end{aligned}$$

(c) Fit the gradient vector $\textbf{U}$ to the parameters by the least squares linear regression procedure. This method is established to be equivalent to the steepest ascent procedure when applied to individual parameters, as detailed in Boyd and Vandenberghe (2004). Moreover, we have expanded this equivalence to encompass groups of parameters. The justification supporting this extension is delineated in the Online Appendix. In this step all the baseline hazard parameter estimates ($\hat{\gamma }$) are updated and one of the other parameters or group of parameters is selected and its estimates updated.

(c.1) Update baseline hazard $\boldsymbol{\gamma }$: At each iteration, the baseline hazard $\boldsymbol{\gamma }$ is updated. The update step $\widetilde{\boldsymbol{\gamma }}^{[m]}$ is determined by the following equation:

5 $$\begin{aligned} \widetilde{\boldsymbol{\gamma }}^{[m]} = \mathop {\text {argmin}}\limits _{\boldsymbol{\gamma }} \sum _{i=1}^n\sum _{s=1}^{T_i}(U_{is}^{[m-1]}-\gamma _s)^2. \end{aligned}$$

Then we update the baseline hazard and $\textbf{U}^{[m-1]}$ as follows:

6 $$\begin{aligned} \widehat{\boldsymbol{\gamma }} ^{[m]}&= \widehat{\boldsymbol{\gamma }} ^{[m-1]} + \nu _{\gamma }\widetilde{\gamma }^{[m]}, \end{aligned}$$

7 $$\begin{aligned} \textbf{U}_{s}^{[m-1]}&\longleftarrow \textbf{U}_{s}^{[m-1]} - \widetilde{\gamma }_s^{[m]}. \end{aligned}$$

The step size $\nu _{\gamma }$ for updating $\gamma$ is set to 1.

*(c.2) Update parameters:* In the same iteration *m*, we select and update one group of parameters choosing between time-independent effects $\boldsymbol{\phi }$, time-varying effects $\boldsymbol{\theta }$, and interaction term effects $\boldsymbol{\alpha }$.

Specifically, the term to be updated, $\hat{\textsf{g}}_m$ is selected based on

8 $$\begin{aligned} \begin{aligned} \hat{\textsf{g}}_m =&\mathop {\text {argmin}}_{\textsf{g}\in \{\textsf{g}_{\text {TV}},\textsf{g}_{\text {TI}},\textsf{g}_{\text {INT}}\},\ \textsf{g}_{\text {TI}} \in \textsf{P}_{\text {TI}},\ \textsf{g}_{\text {TV}} \in \textsf{P}_{\text {TV}},\ \textsf{g}_{\text {INT}} \in G_{2}^{[m]}} \\&\left\{ \text {min}\left( \sum _{i=1}^{n}\sum _{s=1}^{T_i}\left( U_{is}^{[m-1]} - \widetilde{\phi }_{\textsf{g}_{\text {TI}}}x_{i\textsf{g}_{\text {TI}}}\right) ^2, \right. \right. \\&\quad \left. \left. \sum _{i=1}^{n}\sum _{s=1}^{T_i}\left( U_{is}^{[m-1]} - \sum _{k=2}^K\widetilde{\theta }_{\textsf{g}_{\text {TV}}k}B_k(t_s)x_{i\textsf{g}_{\text {TV}}}\right) ^2, \sum _{i=1}^{n}\sum _{s=1}^{T_i}\left( U_{is}^{[m-1]} - \widetilde{\alpha }_{\mathsf {g_{\text {INT}}}}x_{i\mathsf {g_{\text {INT}}}}\right) ^2 \right) \right\} \\ =&\mathop {\text {argmin}}_{\textsf{g}\in \{\textsf{g}_{\text {TV}},\textsf{g}_{\text {TI}},\textsf{g}_{\text {INT}}\},\ \textsf{g}_{\text {TI}} \in \textsf{P}_{\text {TI}},\ \textsf{g}_{\text {TV}} \in \textsf{P}_{\text {TV}},\ \textsf{g}_{\text {INT}} \in G_{2}^{[m]}} \\&\left\{ \text {min}\left( \sum _{i=1}^{n}\sum _{s=1}^{T_i}\left( U_{is}^{[m-1]} - \widetilde{\phi }_{\textsf{g}_{\text {TI}}}x_{i\textsf{g}_{\text {TI}}}\right) ^2, \right. \right. \\&\quad \left. \left. \sum _{i=1}^{n}\sum _{s=1}^{T_i}\left( U_{is}^{[m-1]} - \widetilde{\boldsymbol{\theta }}_{\textsf{g}_{\text {TV}}}^\top \textbf{B}(t_s)x_{i\textsf{g}_{\text {TV}}}\right) ^2, \sum _{i=1}^{n}\sum _{s=1}^{T_i}\left( U_{is}^{[m-1]} - \widetilde{\alpha }_{\mathsf {g_{\text {INT}}}}x_{i\mathsf {g_{\text {INT}}}}\right) ^2 \right) \right\} \end{aligned} \end{aligned}$$

where

$$\begin{aligned} x_{i\textsf{g}} = {\left\{ \begin{array}{ll} z_{i\textsf{g}} & \text {if } \textsf{g} = \textsf{g}_{\text {TI}} \in \textsf{P}_{\text {TI}} = \{1,\ldots ,P\}, \\ z_{i,\textsf{g}-P} & \text {if } \textsf{g} = \textsf{g}_{\text {TV}} \in \textsf{P}_{\text {TV}} = \{P+1,\ldots ,2P\},\\ z_{ij}z_{ij^{'}} & \text {if } \textsf{g} = \textsf{g}_{\text {INT}} = (j,j^{'}) \in \textsf{G}_2^{[m]}, \end{array}\right. } \end{aligned}$$

and

$$\begin{aligned} {\left\{ \begin{array}{ll} \widetilde{\phi }_{\textsf{g}} = \left( \sum \limits _{i=1}^n\sum \limits _{s=1}^{T_i}x_{i\textsf{g}}^2\right) ^{-1}\left( \sum \limits _{i=1}^n\sum \limits _{s=1}^{T_i}x_{i\textsf{g}}U^{[m-1]}_{is}\right) & \text {if } \textsf{g} \in \textsf{P}_{\text {TI}}, \\ \widetilde{\boldsymbol{\theta }}_{\textsf{g}} = \left( \sum \limits _{i=1}^n\sum \limits _{s=1}^{T_i}x_{i,\textsf{g}-P}^2\textbf{B}(t_s)\textbf{B}^{\top }(t_s)\right) ^{-1}\left( \sum \limits _{i=1}^n\sum \limits _{s=1}^{T_i}x_{i,\textsf{g}-P}U^{[m-1]}_{is}\textbf{B}(t_s)\right) & \text {if } \textsf{g} \in \textsf{P}_{\text {TV}}, \\ \widetilde{\alpha }_{\textsf{g}} = \left( \sum \limits _{i=1}^n\sum \limits _{s=1}^{T_i}x_{i\textsf{g}}^2\right) ^{-1}\left( \sum \limits _{i=1}^n\sum \limits _{s=1}^{T_i}x_{i\textsf{g}}U^{[m-1]}_{is}\right) & \text {if } \textsf{g} \in \textsf{G}_2^{[m]}. \end{array}\right. } \end{aligned}$$

The parameter is updated based on the selection results:

9 $$\begin{aligned} {\left\{ \begin{array}{ll} \hat{\phi }_{\textsf{g}} ^{[m]} = \hat{\phi }_{\textsf{g}} ^{[m-1]} + \nu \widetilde{\phi }_{\textsf{g}} & \text {if} \ \textsf{g} \in {\textsf{P}}_{\text {TI}}, \\ \hat{\theta }_{\textsf{g}k} ^{[m]} = \hat{\theta }_{\textsf{g}k} ^{[m-1]} + \nu \widetilde{\theta }_{\textsf{g}k} & \text {if} \ \textsf{g} \in {\textsf{P}}_{\text {TV}}, \\ \hat{\alpha }_{\textsf{g}} ^{[m]} = \hat{\alpha }_{\textsf{g}} ^{[m-1]} + \nu \widetilde{\alpha }_{\textsf{g}} & \text {if} \ \textsf{g} \in {\textsf{G}}_{2}^{[m]}, \\ \end{array}\right. } \end{aligned}$$

where $\nu$ = 0.5. Note that only one parameter or group of parameters will be selected in each iteration.

(c.3) Update the candidate set based on the selected $\hat{\textsf{g}}_{m}$.

$$\begin{aligned} {\left\{ \begin{array}{ll} \textsf{G}_1^{[m]} = \textsf{G}_1^{[m-1]} \cup \{\hat{\textsf{g}}_{m}\} & \text {if } \hat{\textsf{g}}_{m} \in \textsf{p}_{\text {TI}}, \\ \textsf{G}_1^{[m]} = \textsf{G}_1^{[m-1]} \cup \{\hat{\textsf{g}}_{m}-P\} & \text {if } \hat{\textsf{g}}_{m} \in \textsf{p}_{\text {TV}}, \\ \textsf{G}_1^{[m]} = \textsf{G}_1^{[m-1]} & \text {if } \hat{\textsf{g}}_{m-1} \in \textsf{G}_2^{[m]}, \end{array}\right. } \end{aligned}$$

and correspondingly $\textsf{G}_2^{[m]} = \{(j,j^{'}):\ j,j^{'}\in \textsf{G}_1^{[m]} \}$. Note that the candidates set is updated if one main effect is selected for the first time (either time-independent or time-varying).

(d) Stop the algorithm when $m = m_{stop}$. Choosing an appropriate $m_{stop}$ is important; existing methods for its selection are often ad-hoc, and early stopping is a common technique. We consider two possible approaches. One is based on adapting ideas related to BIC and degrees of freedom for boosting algorithms and is described in Sect. 2.4. The other is based on re-estimation of the selected model (described in Sect. 2.5) and calculation of the degrees of freedom to use in BIC. This approach to the calculation of the degrees of freedom is described in Sect. 2.6. A step-by-step summary of the proposed DiscBoosting algorithm is provided in Online Appendix Sect. 2.5.

### Boosting algorithm degrees of freedom

To determine a stopping iteration $m_{stop}$, one approach is to calculate the approximate ‘hat’ matrix $\mathcal {B}_m$ to compute the degrees of freedom, which is defined as trace($\mathcal {B}_m$) (Bühlmann and Yu 2006). This calculation involves the $\mathcal {H}^{\hat{\textsf{g}}}$ matrix and the diagonal matrix *W* (definitions provided in the Online Appendix). The iterative equations for $\mathcal {B}_m$ are:

$$\begin{aligned} \mathcal {B}_1&= \nu W^{[0]}\mathcal {H}^{\hat{\textsf{g}}_0}, \\ \mathcal {B}_m&= \mathcal {B}_{m-1} + \nu W^{[m-1]}\mathcal {H}^{\hat{\textsf{g}}_{m}}(I-\mathcal {B}_{m-1})\ (m\ge 2). \end{aligned}$$

The stopping step is determined by calculating the BIC at each iteration, as expressed by the following equation:

10 $$\begin{aligned} \text {BIC}(m) = -2\ell + \log (n)\text {df}(m), \end{aligned}$$

where the degrees of freedom is given by

11 $$\begin{aligned} \text {df}(m)&= \text {trace}{(\mathcal {B}_m)} , \end{aligned}$$

and the algorithm stops at the value of *m* that minimizes $\text {BIC}(m)$.

### Newton’s method for discrete survival model with time-varying effects and interaction terms

The boosting procedure outlined in Sect. 2.3 offers a powerful tool for variable selection in high-dimensional settings. However, it may lead to underestimation of effect sizes, in particular parameter estimates are frequently too close to zero. To mitigate this limitation and achieve more stable estimation, we leverage the variables selected by DiscBoosting and re-fit the model by applying Newton’s method tailored for discrete survival models with time-varying effects and interaction terms. Using the notation introduced in Sect. 2.1, we modify the B-spline basis $\boldsymbol{B}(t)$ following the standard parameterization described in He et al. (2021). Let the full parameter vector be denoted by $\boldsymbol{\omega } = (\boldsymbol{\gamma }, \boldsymbol{\phi }, \boldsymbol{\theta }, \boldsymbol{\alpha })$. The corresponding penalized log-likelihood to be maximized is

12 $$\begin{aligned} \ell _\lambda (\boldsymbol{\omega }) = \ell (\boldsymbol{\omega }) - \frac{1}{2} P_\lambda (\boldsymbol{\theta }) = \ell (\boldsymbol{\omega }) - \frac{1}{2}\lambda \boldsymbol{\theta }^T \textbf{S}\boldsymbol{\theta }, \end{aligned}$$

where $\ell (\boldsymbol{\omega })$ is the same as formula 3. For the penalty function $P_\lambda (\boldsymbol{\theta })$, and the corresponding matrix $\textbf{S}$ common choices are discrete penalties and derivative based penalties, which we call P-spline and Smoothing-Spline. Details of these penalties are provided in the Online Appendix. The smoothing parameter $\lambda$ controls the amount of smoothness of the time-varying coefficients, and we need to select an optimal value for it. For a given $\lambda$ the parameter estimates $\hat{\theta }_\lambda$ are obtained by maximizing (12). In this paper the selection of $\lambda$ is determined by Takeuchi information criteria (TIC) (Luo et al. 2023).

For a fixed value of $\lambda$, maximization of the penalized log likelihood (12) can be achieved by Newton’s method, which requires computation of the gradient and information matrix, given by $\nabla \ell _\lambda (\boldsymbol{\theta }) = \nabla \ell (\boldsymbol{\theta }) - \lambda \textbf{S}\boldsymbol{\theta }$ and $I_\lambda (\boldsymbol{\theta }) = I_{0}(\boldsymbol{\theta }) + \lambda \textbf{S}$, where $I_{0}(\boldsymbol{\theta })$ is the negative of the Hessian matrix $\nabla ^2 \ell (\boldsymbol{\theta })$. (Detailed derivations of the gradient and information matrix for our method and how they can be efficiently calculated are provided in the Online Appendix). The algorithm does not require reorganizing the data into a long format, making it applicable to datasets with large sample sizes. In the algorithm the current value of $\boldsymbol{\theta }$ is updated using the step:

13 $$\begin{aligned} \boldsymbol{\theta }_{new} = \boldsymbol{\theta }_{old} - \alpha (\nabla ^2 \ell _\lambda (\boldsymbol{\theta }_{old}))^{-1} \nabla \ell _\lambda (\boldsymbol{\theta }_{old}) \end{aligned}$$

In a standard Newton algorithm $\alpha =1$, but we use an adaptive choice of $\alpha \le 1$ via a backtracking line search to improve convergence (Boyd and Vandenberghe 2004).

### Parameter re-estimation based stopping

In section 2.4 we defined the degrees of freedom in the boosting framework. However, the calculation of the ‘hat’ matrix is necessary at each iteration and also requires a large memory. We also found that the parameter estimates from the boosting algorithm could exhibit considerable bias and variability. Thus, we considered a different way of calculating BIC from the selected variables. Motivated by the “degrees of freedom” concept as described in Hastie and Tibshirani (1990), we refit a discrete failure time model with the selected covariates at iteration step *m* in the boosting algorithm using the maximum penalized likelihood parameter estimates, as described in Section 2.5, and calculate the “degrees of freedom” from the fitted model:

$$\begin{aligned} \text {df}(m)&= tr(2{\hat{I}_{\lambda }}^{-1}(m)\hat{I}_0(m) - \hat{I}_0(m){\hat{I}_{\lambda }}^{-1}(m)\hat{I}_0(m){\hat{I}_{\lambda }}^{-1}(m)), \end{aligned}$$

where $I_{0}(m)$ is the negative of the Hessian matrix $\nabla ^2 \ell (\boldsymbol{\theta })$. To determine the optimal stopping iteration $m_{stop}$, we replace the degrees of freedom in Eq. 11 for BIC with the one calculated here, and choose the value of *m* that minimizes *BIC*(*m*). Furthermore, for the scenarios we considered we found that it is not necessary to refit the model at every iteration. We observed that the results barely changed when we calculated BIC at every 5th iteration, thus also enhancing computational efficiency.

## Simulation studies

Simulation studies were conducted to evaluate the performance of the proposed method. First, we demonstrated its properties in terms of variable selection of both constant and time-varying effects in high-dimensional settings without interactions. Our aim was to determine if the method could accurately (1) identify overall true signals and (2) correctly distinguish between important variables with time-varying and time-independent effects. Secondly, the accuracy of the estimates of the time-varying coefficient curves (the $\beta _j(t)$’s), the interaction coefficients and the baseline hazard parameters were evaluated. Finally, we evaluate the impact of imposing the hierarchy restrictions on the selection of interactions.

### High dimensional variable selection

In the simulations for high-dimensional variable selection, the continuous covariates $\boldsymbol{Z}$ were generated from a multivariate normal distribution with zero mean, unit variance and a first-order autoregressive structure with the auto-correlation parameter 0.5. In addition, to consider a more challenging situation, we transformed the continuous variables into binary variables (coded as 0 or 1), with the probability of being zero uniformly varying from 0.05 to 0.15. Failure times were generated from a discrete logistic model with hazard function as

$$\begin{aligned} \lambda (t_s;{\textbf {Z}}_{i})=\frac{\exp \{(\gamma _s + {\textbf {Z}}^\top _{i} \boldsymbol{\beta }(t_s))\}}{1+\exp {\{(\gamma _s + {\textbf {Z}}^\top _{i} \boldsymbol{\beta }(t_s))\}}}, \end{aligned}$$

where $$\gamma _s = \{-4.00, -3.50, -3.20,$$$$-3.12, -3.08, -3.00,$$ $$-2.95, -2.87, -2.80,$$ $$-2.69,$$ $$-2.61,$$ $$-2.49,$$ $$-2.39,$$ $$-2.25,$$ $$-2.10,$$ $$-1.90,$$ $$-1.70, -1.35, -1.00\}$$. Five covariates have non-zero $$\beta _j(t)$$ coefficients: specifically, $$\beta _1(t)=1$$, $$\beta _2(t) = \cos (\pi t/50)$$, $$\beta _3(t) = -1$$, $$\beta _4(t) = \sin (3\pi t/80)$$, and $$\beta _5(t) = -1+\exp (-0.25t)$$. Censoring times were generated from a discrete uniform distribution. The number of subjects was 500, and the total number of covariates was set to 100, 500 or 1000. 

**Fig. 1:**
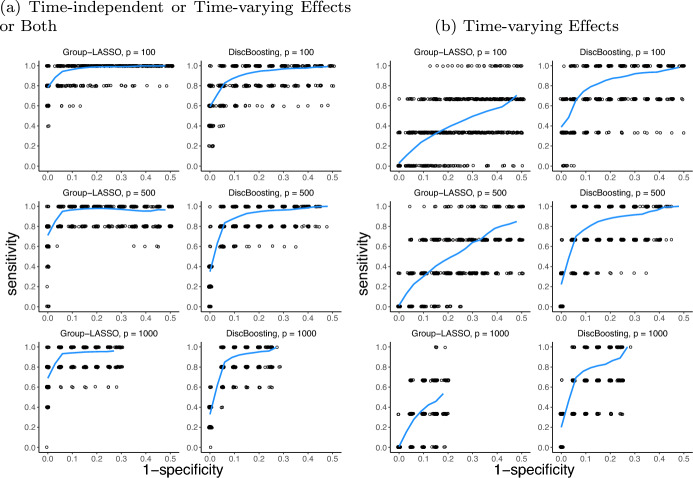
This plot illustrates the sensitivity and specificity of selected covariates through simulation. With a sample size of 500, the number of covariates is varied, taking values of 100, 500, and 1000. The non-zero components of the covariate vector are $\beta _1(t)=1$, $\beta _2(t) = \cos (\pi t/50)$, $\beta _3(t) = -1$, $\beta _4(t) = \sin (3\pi t/80)$, $\beta _5(t) = -1+\exp (-0.25t)$. **a** Performance of variable selection for either time-independent or time-varying effects or both. **b** Specifically assesses the selection of time-varying effects. By altering the penalization coefficients assigned to the Group-LASSO and adjusting the number of iteration steps used in DiscBoosting, a variety of sensitivity and specificity combinations are achieved for each data replicate. Each of these combinations is denoted by a dot on the plot. Each data replicate contributes many dots in the plot. A LOESS smoother is employed for clear representation

To perform variable selection in the presence of time-varying coefficients, an alternative to the proposed boosting method is a Group Lasso-type procedure (Yan and Huang 2012; Möst and Pößnecker 2016). Table 1 and Fig. 1 show the performance of our proposed method by comparing it to Group-LASSO. We generated 100 independent data replicates for each configuration. Table 1 reports six measures: the average false positive (FP) count, average false negative (FN) count, empirical probability of correctly identified informative predictors (SE), the ratio of correctly ignored noise to the total noise count (SP), positive predictive value (PPV), and negative predictive value (NPV). In Fig. 1, we varied the penalization coefficients of the Group-LASSO and the number of iteration steps used in the DiscBoosting, resulting in different combinations of sensitivity and specificity for each data replicate. Each combination is represented by a dot on the plot and a LOESS smoother is used for clear comparison.

**Table 1:** Evaluation of variable selection performance

Number of covariates	Variable	Measure	Method	Stopping criteria	FP (sd)	FN (sd)	SE (sd)	SP (sd)	PPV (sd)	NPV (sd)
P = 100	Continuous	TI or TV	DiscBoosting	Boosting BIC	7.35 (2.03)	0.13 (0.37)	0.97 (0.07)	0.92 (0.02)	0.41 (0.07)	1.00 (0.00)
				Parameter re-estimation BIC	0.24 (0.61)	0.80 (0.61)	0.84 (0.12)	1.00 (0.01)	0.96 (0.09)	0.99 (0.01)
			Group-LASSO	–	16.83 (7.00)	0.00 (0.00)	1.00 (0.00)	0.82 (0.07)	0.26 (0.09)	1.00 (0.00)
		TV	DiscBoosting	Boosting BIC	8.41 (2.02)	0.46 (0.59)	0.85 (0.20)	0.91 (0.02)	0.24 (0.07)	0.99 (0.01)
				Parameter re-estimation BIC	1.36 (1.06)	1.33 (0.85)	0.56 (0.28)	0.99 (0.01)	0.60 (0.27)	0.99 (0.01)
			Group-LASSO	–	8.29 (7.94)	2.04 (0.84)	0.32 (0.28)	0.91 (0.08)	0.24 (0.33)	0.98 (0.01)
	Binary	TI or TV	DiscBoosting	Boosting BIC	3.01 (1.51)	1.32 (0.85)	0.74 (0.17)	0.97 (0.02)	0.57 (0.14)	0.99 (0.01)
				Parameter re-estimation BIC	0.72 (0.88)	1.67 (0.89)	0.67 (0.18)	0.99 (0.01)	0.86 (0.16)	0.98 (0.01)
			Group-LASSO	–	17.12 (8.08)	0.10 (0.33)	0.98 (0.07)	0.82 (0.09)	0.25 (0.09)	1.00 (0.00)
		TV	DiscBoosting	Boosting BIC	4.62 (1.53)	1.23 (0.78)	0.59 (0.26)	0.95 (0.02)	0.28 (0.12)	0.99 (0.01)
				Parameter re-estimation BIC	2.31 (1.05)	1.52 (0.76)	0.49 (0.25)	0.98 (0.01)	0.39 (0.16)	0.98 (0.01)
			Group-LASSO	–	3.31 (5.46)	2.78 (0.52)	0.07 (0.17)	0.97 (0.06)	0.44 (0.48)	0.97 (0.01)
P = 500	Continuous	TI or TV	DiscBoosting	Boosting BIC	15.07 (3.53)	0.27 (0.45)	0.95 (0.09)	0.97 (0.01)	0.25 (0.04)	1.00 (0.00)
				Parameter re-estimation BIC	0.45 (0.80)	0.91 (0.55)	0.82 (0.11)	1.00 (0.00)	0.93 (0.12)	1.00 (0.00)
			Group-LASSO	–	27.92 (14.67)	0.05 (0.22)	0.99 (0.04)	0.94 (0.03)	0.18 (0.08)	1.00 (0.00)
		TV	DiscBoosting	Boosting BIC	16.01 (3.32)	0.75 (0.73)	0.75 (0.24)	0.97 (0.01)	0.13 (0.04)	1.00 (0.00)
				Parameter re-estimation BIC	1.56 (0.98)	1.47 (0.88)	0.51 (0.29)	1.00 (0.00)	0.51 (0.25)	1.00 (0.00)
			Group-LASSO	–	1.48 (3.92)	2.85 (0.44)	0.05 (0.15)	1.00 (0.01)	0.63 (0.47)	0.99 (0.00)
	Binary	TI or TV	DiscBoosting	Boosting BIC	5.76 (1.98)	1.76 (0.85)	0.65 (0.17)	0.99 (0.00)	0.37 (0.10)	1.00 (0.00)
				Parameter re-estimation BIC	1.92 (1.73)	2.08 (0.90)	0.58 (0.18)	1.00 (0.00)	0.67 (0.21)	1.00 (0.00)
			Group-LASSO	–	21.46 (13.52)	0.39 (0.57)	0.92 (0.11)	0.96 (0.03)	0.23 (0.13)	1.00 (0.00)
		TV	DiscBoosting	Boosting BIC	7.25 (2.00)	1.55 (0.74)	0.48 (0.25)	0.99 (0.00)	0.17 (0.09)	1.00 (0.00)
				Parameter re-estimation BIC	3.31 (1.90)	1.78 (0.76)	0.41 (0.25)	0.99 (0.00)	0.28 (0.17)	1.00 (0.00)
			Group-LASSO	–	0.41 (1.26)	2.97 (0.17)	0.01 (0.06)	1.00 (0.00)	0.81 (0.39)	0.99 (0.00)
P = 1000	Continuous	TI or TV	DiscBoosting	Boosting BIC	20.52 (4.26)	0.30 (0.46)	0.94 (0.09)	0.98 (0.00)	0.19 (0.03)	1.00 (0.00)
				Parameter re-estimation BIC	0.58 (1.10)	1.02 (0.57)	0.80 (0.11)	1.00 (0.00)	0.91 (0.15)	1.00 (0.00)
			Group-LASSO	–	31.77 (14.31)	0.05 (0.22)	0.99 (0.04)	0.97 (0.01)	0.16 (0.07)	1.00 (0.00)
		TV	DiscBoosting	Boosting BIC	21.22 (4.15)	0.71 (0.74)	0.76 (0.25)	0.98 (0.00)	0.10 (0.03)	1.00 (0.00)
				Parameter re-estimation BIC	1.74 (1.42)	1.47 (0.83)	0.51 (0.28)	1.00 (0.00)	0.52 (0.27)	1.00 (0.00)
			Group-LASSO		0.58 (1.71)	2.96 (0.20)	0.01 (0.07)	1.00 (0.00)	0.73 (0.44)	1.00 (0.00)
	Binary	TI or TV	DiscBoosting	Boosting BIC	8.00 (3.19)	2.09 (0.87)	0.58 (0.17)	0.99 (0.00)	0.28 (0.09)	1.00 (0.00)
				Parameter re-estimation BIC	3.02 (2.44)	2.52 (0.90)	0.50 (0.18)	1.00 (0.00)	0.52 (0.23)	1.00 (0.00)
			Group-LASSO	–	21.17 (13.49)	0.59 (0.75)	0.88 (0.15)	0.98 (0.01)	0.23 (0.13)	1.00 (0.00)
		TV	DiscBoosting	Boosting BIC	9.34 (3.23)	1.80 (0.75)	0.40 (0.25)	0.99 (0.00)	0.12 (0.07)	1.00 (0.00)
				Parameter re-estimation BIC	4.27 (2.55)	2.08 (0.76)	0.31 (0.25)	1.00 (0.00)	0.19 (0.16)	1.00 (0.00)
			Group-LASSO	–	0.09 (0.35)	3.00 (0.00)	0.00 (0.00)	1.00 (0.00)	0.93 (0.26)	1.00 (0.00)

False positive (FP) was the number of predictors selected by the algorithms, when the true effect was zero. True positive (TP) was the number of predictors correctly identified. False negative (FN) was the number of true signals that the algorithms didn’t select. True negative (TN) was the number of noises correctly identified as having no effect. Sensitivity (SE) was calculated using the number of correctly chosen signals divided by the number of true signals. Specificity (SP) was calculated using the number of correctly ignored noises divided by the number of noises. Positive predictive value (PPV) was calculated using TP divided by the number of selected predictors. Negative predictive value (NPV) was calculated using TN divided by the number of ignored predictors. The sample size is 500 patients with the number of covariates set at 100, 500, and 1000. Each simulation scenario was replicated 100 times. The covariate vector’s non-zero components were defined as follows: $\beta _1(t)=1$, $\beta _2(t) = \cos (\pi t/50)$, $\beta _3(t) = -1$, $\beta _4(t) = \sin (3\pi t/80)$, $\beta _5(t) = -1+\exp (-0.25t)$. Both continuous and binary covariates were included in the analysis. The ‘TI or TV’ metric assesses the algorithms’ ability to select either time-independent or time-varying effects or both accurately, whereas ‘TV’ focuses on the selection of time-varying effects. The proposed boosting method was evaluated using two different stopping criteria: the Boosting BIC and parameter re-estimation BIC. These were compared with the performance of Group-LASSO

In terms of variable selection performance for either time-varying effects or time-independent effects or both, while Group-LASSO identified more informative variables, this increment was offset by an increase in FN. While in detecting time-varying effects, Group-LASSO encountered challenges, evidenced by a higher rate of FN, indicating difficulty in accurately identifying time-varying effects.

In Sect. 3.3 in the Online Appendix we demonstrate that the DiscBoosting algorithm is computationally efficient and scalable compared to Group-LASSO in this setting.

### Estimation accuracy after variable selection for models with interactions

In this section we consider the situation of a more modest number of covariates and allow for all possible 2-way interactions. We consider the bias and variability of the selected model, where the selected model is determined by the main effects, time-varying effects and interactions (under strong hierarchical constraints), at the time the algorithm stopped. The simulation study was conducted with a sample size of $$n = 1000$$ or $$n = 4000$$ and $$P = 15$$ predictors. Both training and testing datasets were generated. The total number of possible parameters (main effects plus interactions plus spline terms plus baseline hazards) was 230. The predictors $$\textbf{Z}$$ were generated from a multivariate normal distribution with zero mean, unit variance and a first-order autoregressive structure with the auto-correlation parameter 0.5. Failure times were generated from a discrete logistic model with covariate effects as $$\boldsymbol{\beta }(t)$$ and baseline hazards as $$\boldsymbol{\gamma }_k$$, where $$\gamma _k$$ = {- 4.3, - 4.05, - 3.8, - 3.5, - 3.12, - 2.78, - 2.32, - 2.12, - 1.99, - 1.8, - 1.69, - 1.61, - 1.49, - 1.39, - 1.25, - 1.1, - 0.9, - 0.7, - 0.35, 0}. We consider the setting where the coefficients of most main effects are non-zero. The covariates with time-varying effects are $$\beta _1(t) = 1 + \cos (\pi t/50), \beta _2(t) = -1 + \exp (-0.25t), \beta _3(t) = 1-\cos (\pi t/30), \beta _4(t) = \sin (3\pi t/80),$$ and $$\beta _5(t) = -\sin (\pi t/30)$$. The remaining 10 covariates have time-independent or zero effects equal to $$(-1,-1,-1,1,1,1,1,0,0,0)$$. The following interaction terms $$z_1z_2, z_1z_3, z_1z_9, z_5z_{10}, z_6z_7$$ have non-zero coefficients equal to $$(1,1,1,$$ $$-1,$$ $$-1)$$. Censoring times were generated from a discrete uniform distribution.

We evaluate two different stopping criteria as discussed in Sects. 2.4 and 2.6, referred to as Boosting BIC and parameter re-estimation BIC respectively. Four specific scenarios are considered:

- Full model: All the covariates are incorporated in the model as time-varying effects; all the possible two-way interactions are incorporated as time-independent effects.
- Boosting BIC: Covariates are incorporated in the model based on DiscBoosting selection results with Boosting BIC as the stopping criterion.
- Re-estimation BIC: Covariates are incorporated in the model based on DiscBoosting selection results with parameter re-estimation BIC as the stopping criterion.
- Benchmark: Only covariates with true signals are incorporated into the model. Note that this ideal setting serves as a benchmark and is not known in the context of real data.

**Fig. 2:**
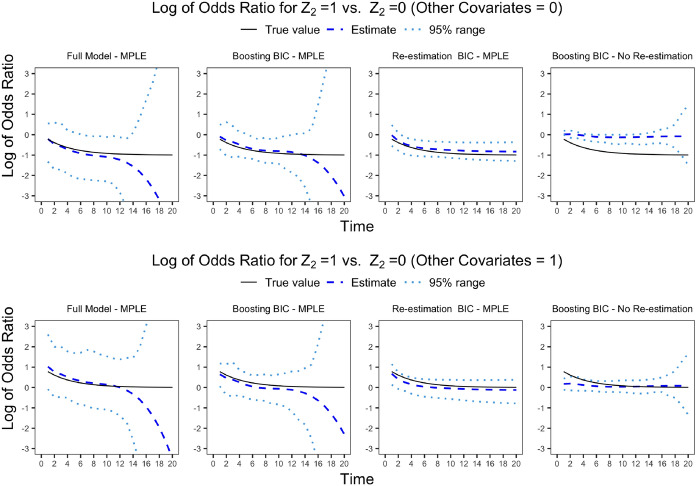
This figure presents the log of odds ratio (dashed lines) for $Z_2=1$ vs. $Z_2=0$ across time points *t*. The 95% empirical percentile range for the log odds ratio is shown by dotted lines (2.5% and 97.5% as lower and upper bounds). True values are plotted in solid lines. In each scenario, 100 data replicates were generated with a sample size of 1000. The model was fitted using a fixed number of $K=7$ basis functions. True values were $\beta _1(t) = -1 + \cos (\pi t/10)$, $\beta _2(t) = -1 + \exp (-0.25t)$, $\beta _3(t) = sin(\pi t/10), \beta _4(t) = 1, \beta _5(t) = -1$. The top plot is the log of odds ratio for a unit change in $Z_2$, given $(Z_1,Z_3,\ldots ,Z_P) = (0,0,\ldots ,0)$, and the bottom plot corresponds to $(Z_1,Z_3\ldots ,Z_P) =(1,1,\ldots ,1)$. We evaluated four distinct different approaches to obtain the estimates: Full model, where all covariates are treated as time-varying and all two-way interactions are time-independent; Boosting BIC, with covariates selected via DiscBoosting using the Boosting BIC stopping criterion; Re-estimation BIC, where DiscBoosting with parameter re-estimation BIC as the stopping criterion is used for variable selection; and DiscBoosting estimation results. For the first three methods, the estimation is based on maximized penalized likelihood using P-splines, where the penalization coefficient of P-spline is chosen by TIC. DiscBoosting estimate is based on Boosting BIC stopping criterion without re-estimation

Table 2 presents various metrics including average bias, integrated mean squared error (IMSE), average standard deviation (SD), for the estimated main effects $(\phi _j + \beta _j(t))$, the interaction terms $(\alpha _{j,j'})$ and the baseline hazards $(\gamma _s)$ with different methods. Furthermore, Table S6 in the Online Appendix provides the negative log-likelihood for both training and testing datasets, as well as the bias, IMSE, and SD for overall model performance. The definitions of bias, IMSE and SD are also given in the Online Appendix. The selection of covariates included in the models was based on the four outlined scenarios. Discrete failure time models were fitted employing Newton’s method to obtain the estimates. The performance between non-penalized, P-spline, and Smoothing-spline estimation approaches were evaluated. Figure 2 illustrates the variation in the odds ratio associated with a unit increase in $Z_2$, while setting other covariates as 0 and 1. It includes both point estimates and the corresponding 95 percentiles of the empirical distribution of the point estimates. Figure 2 shows that the full model gives the most variable estimates of the time-varying coefficient curves, that gradient boosting procedures reduce the variability, that the parameters estimates that are given directly by the boosting algorithm are biased towards zero, and that this bias is removed by parameter re-estimation. Stopping the algorithm based on parameter re-estimation BIC is better at reducing the variability than that from the boosting based BIC. The results for the log-likelihood on the training and testing data also show reduction in over-fitting due to the combination of gradient boosting coordinate-wise variable selection, the penalized likelihood model fitting and the use of parameter re-estimation based BIC to stop the algorithm.

**Table 2:** Estimation results for covariate effects: bias, IMSE, SD for main effects, interactions and baseline hazard

Evaluation	Scenario	Estimation	Bias	SD	IMSE
Main effects	Benchmark	NR (no penalty)	16.50	21.10	0.70
		NR (P-spline)	13.40	16.40	0.30
		NR (Smooth-spline)	14.60	18.30	0.50
	Full model	NR (no penalty)	57.41	69.30	6.50
		NR (P-spline)	35.53	41.22	1.91
		NR (Smooth-spline)	44.26	51.95	3.16
	Boosting BIC	NR (no penalty)	33.97	39.88	2.40
		NR (P-spline)	27.65	26.17	1.37
		NR (Smooth-spline)	30.23	32.03	1.70
		No Re-estimation	63.49	12.27	5.40
	Re-estimation BIC	NR (P-spline)	26.71	23.73	1.33
		NR (Smooth-spline)	27.52	24.93	1.41
Interactions	Benchmark	NR (no penalty)	0.85	1.08	0.16
		NR (P-spline)	0.84	1.05	0.16
		NR (Smooth-spline)	0.83	1.05	0.16
	Full model	NR (no penalty)	24.30	30.43	6.27
		NR (P-spline)	22.23	27.42	5.23
		NR (Smooth-spline)	23.26	28.76	5.75
	Boosting BIC	NR (no penalty)	5.75	8.30	2.09
		NR (P-spline)	5.72	8.00	2.08
		NR (Smooth-spline)	5.67	8.10	2.05
		No Re-estimation	6.66	3.50	2.73
	Re-estimation BIC	NR (P-spline)	5.31	6.32	2.08
		NR (Smooth-spline)	5.35	6.44	2.10
Baseline hazard	Benchmark	NR (no penalty)	34.58	47.38	0.27
		NR (P-spline)	28.63	35.02	0.10
		NR (Smooth-spline)	30.43	36.78	0.12
	Full model	NR (no penalty)	63.99	79.78	0.57
		NR (P-spline)	41.56	49.76	0.19
		NR (Smooth-spline)	51.11	60.99	0.28
	Boosting BIC	NR (no penalty)	54.69	64.77	0.52
		NR (P-spline)	44.53	43.08	0.21
		NR (Smooth-spline)	46.01	48.42	0.27
		No Re-estimation	59.73	34.67	0.35
	Re-estimation BIC	NR (P-spline)	41.18	41.82	0.19
		NR (Smooth-spline)	42.82	41.84	0.20

The sample size is 1000 and there are 15 covariates, of which 5 have time-varying effects, 7 have time-independent effects and 3 have no effect. We evaluated four distinct scenarios: Full model, where all covariates were treated as time-varying and all two-way interactions are included; Boosting BIC, with covariates selected via DiscBoosting using the Boosting BIC stopping criterion; Re-estimation BIC, where DiscBoosting with parameter re-estimation BIC as the stopping criterion was used for covariate selection; and Benchmark, an idealized scenario incorporating only covariates with true signals for comparison purposes. No Re-estimation refers to boosting estimation. All reported Bias, SD and IMSE values are multiplied by 100 for readability

Table 2 demonstrates that the Full model, which didn’t employ variable selection, consistently exhibited higher bias, SD, IMSE, for all of the parameters. Again the combination of gradient boosting coordinate-wise variable selection, the penalized likelihood model fitting and the use of parameter re-estimation based BIC to stop the algorithm lead to the best bias, SD and IMSE. Table S6 in the Online Appendix shows similar results for n = 4000. All the above results are for odds ratios, but also apply to the time-varying hazard ratio curves, when calculated using Eq. 2.

### Hierarchical conditions for interactions

The DiscBoosting algorithm has flexibility to accommodate different hierarchical structures. This subsection evaluates the variable selection performance of the proposed boosting algorithm under various hierarchical constraints for the interactions. The simulation setup follows the strong hierarchical structure described in Sect. 3.2.

Table 3 displays the False Positive (FP), False Negative (FN), Sensitivity (SE), Specificity (SP), Positive Predictive Value (PPV) and Negative Predictive Value (NPV) metrics. Results indicate that the algorithm performs well under a strong hierarchy structure. However, even when there is a mis-specification in the selected method, the variable selection performance remains relatively stable.

**Table 3:** The effect of imposing hierarchy constraints on selection of interactions

Stopping criteria	Measure	Hierarchy	FP (sd)	FN (sd)	SE (sd)	SP (sd)	PPV (sd)	NPV (sd)
Boosting BIC	TI or TV	Strong hierarchy	30.27 (4.17)	0.03 (0.17)	1.00 (0.01)	0.71 (0.04)	0.36 (0.03)	1.00 (0.00)
		Weak hierarchy	32.15 (3.73)	0.02 (0.14)	1.00 (0.01)	0.69 (0.04)	0.35 (0.03)	1.00 (0.00)
		No hierarchy	31.82 (3.69)	0.02 (0.14)	1.00 (0.01)	0.69 (0.04)	0.35 (0.03)	1.00 (0.00)
	TV	Strong hierarchy	8.92 (0.92)	0.02 (0.14)	1.00 (0.03)	0.92 (0.01)	0.36 (0.03)	1.00 (0.00)
		Weak hierarchy	8.74 (1.02)	0.00 (0.00)	1.00 (0.00)	0.92 (0.01)	0.37 (0.03)	1.00 (0.00)
		No hierarchy	8.72 (1.06)	0.01 (0.10)	1.00 (0.02)	0.92 (0.01)	0.37 (0.03)	1.00 (0.00)
	Interactions	Strong hierarchy	28.11 (3.65)	0.03 (0.17)	0.99 (0.03)	0.72 (0.04)	0.15 (0.02)	1.00 (0.00)
		Weak hierarchy	30.07 (3.62)	0.02 (0.14)	1.00 (0.03)	0.70 (0.04)	0.14 (0.02)	1.00 (0.00)
		No hierarchy	29.72 (3.61)	0.02 (0.14)	1.00 (0.03)	0.70 (0.04)	0.14 (0.01)	1.00 (0.00)
Parameter re-estimation BIC	TI or TV	Strong hierarchy	22.13 (3.30)	0.12 (0.33)	0.99 (0.02)	0.79 (0.03)	0.44 (0.03)	1.00 (0.00)
		Weak hierarchy	19.17 (2.16)	2.03 (1.11)	0.88 (0.07)	0.81 (0.02)	0.44 (0.03)	0.98 (0.01)
		No hierarchy	19.16 (2.17)	2.07 (1.39)	0.88 (0.08)	0.81 (0.02)	0.44 (0.04)	0.98 (0.02)
	TV	Strong hierarchy	7.06 (1.54)	0.07 (0.26)	0.99 (0.05)	0.94 (0.01)	0.42 (0.06)	1.00 (0.00)
		Weak hierarchy	4.67 (1.51)	0.84 (0.87)	0.83 (0.17)	0.96 (0.01)	0.48 (0.08)	0.99 (0.01)
		No hierarchy	4.64 (1.57)	0.85 (0.86)	0.83 (0.17)	0.96 (0.01)	0.48 (0.08)	0.99 (0.01)
	Interactions	Strong hierarchy	21.37 (2.72)	0.12 (0.33)	0.98 (0.07)	0.79 (0.03)	0.19 (0.02)	1.00 (0.00)
		Weak hierarchy	19.14 (2.14)	1.33 (0.53)	0.73 (0.11)	0.81 (0.02)	0.16 (0.02)	0.98 (0.01)
		No hierarchy	19.13 (2.17)	1.28 (0.49)	0.74 (0.10)	0.81 (0.02)	0.16 (0.02)	0.98 (0.01)

False positive (FP) was the number of predictors selected by the algorithms, when the true effect was zero. True positive (TP) was the number of predictors correctly identified. False negative (FN) was the number of true signals that the algorithms didn’t select. True negative (TN) was the number of noises correctly identified as having no effect. Sensitivity (SE) was calculated using the number of correctly chosen signals divided by the number of true signals. Specificity (SP) was calculated using the number of correctly ignored noises divided by the number of noises. Positive predictive value (PPV) was calculated using TP divided by the number of selected predictors. Negative predictive value (NPV) was calculated using TN divided by the number of ignored predictors. The model is fitted using a fixed number of $K=7$ basis functions. The covariates with time-varying effects are $\beta _j(t) = (1 + \cos (\pi t/50), -1 + \exp (-0.25t), 1-\cos (\pi t/30), \sin (3\pi t/80), -\sin (\pi t/30)), j = 1,\dots ,5$. The remaining 10 main covariates have time-independent effects with $\beta _j(t) = (-1,-1,-1,1,1,1,1,0,0,0), j = 6,\dots ,15$. The following interaction terms $z_1z_2, z_1z_3, z_1z_9, z_5z_{10}, z_6z_7$ have non-zero effects with $(1,1,1,-1,-1)$. The sample size is 1,000. The ‘TI or TV’ metric assesses the algorithms’ ability to select either time-independent or time-varying effects or both accurately ‘TV’ focuses on time-varying effects, and ‘Interactions’ metric evaluates the correct identification of interaction terms. The performance of the DiscBoosting algorithm is evaluated across three user-specified hierarchical constraints: strong hierarchy, weak hierarchy, and no hierarchy

In this simulation setting, interaction term effects are present only when corresponding main effects also included. When our method is set to a strong hierarchy in this context, it aligns well with the setup and yields comparable outcomes for both Boosting BIC and parameter re-estimation BIC stopping criteria. However, when the method is configured to employ either a weak hierarchy or no hierarchy structure, it deviates from the simulation’s inherent strong hierarchy. In such cases, especially with the earlier stopping by parameter re-estimation BIC, we observe an increase in FN and a decrease in NPV, reflecting the sensitivity of the method to the hierarchical structure imposed by the user.

In the Online Appendix, we include several additional sets of simulations to further assess the DiscBoosting procedure’s performance. Section 3.1 evaluates the method’s robustness under higher signal densities by increasing the number of true signals to 20 and 50 in high-dimensional settings. Section 3.2 presents a comprehensive evaluation of the algorithm’s ability to simultaneously select main effects, time-varying effects, and interaction terms in an expanded high-dimensional scenario involving 40 main covariates and 780 possible interactions. Section 3.3 demonstrates the computational efficiency and linear scalability of DiscBoosting compared to Group LASSO as sample size and dimensionality increase. Section 3.4, based on fitted results from the melanoma cancer analysis, demonstrates the procedure’s reliable performance in a different but realistic data generating scenario. Section 3.5 highlights the procedure’s ability to incorporate time-varying hierarchical conditions. This leads to improved variable selection accuracy, particularly in high-dimensional settings.

## Analysis of melanoma cancer survival data

To demonstrate the performance of the proposed procedures, we applied the methods to melanoma cancer survival data in the SEER registry. In recent years clinical trials have shown that immunotherapy is an effective treatment for metastatic melanoma and the usage of immunotherapy has increased over time. Because of this we might expect to see reduced hazards of death due to cancer over time and a question of interest is whether these improvements are similar in different subgroups of patients. Thus we are particularly interested in interactions between covariates that represent calendar time and covariates that define patient subgroups.

Patients first diagnosed with metastatic melanoma between 2004 and 2017 were considered for the analysis. The covariates considered were age, race, sex and year of diagnosis. Each covariate was treated as a categorical variable: age was categorized into four levels, race into four levels, and year of diagnosis into three levels. The SEER registry provides the cause of death for each patient, distinguishing between deaths due to cancer and those due to other causes. We employed the proposed DiscBoosting procedure with parameter re-estimation BIC as the stopping criterion to identify time-independent and time-varying covariates, as well as significant interactions, for the outcome of death due to cancer. The analysis was conducted on a dataset comprising 10,912 cases of metastatic melanoma.

Using the boosting procedure, it was found that the variables age under 50, age over 70, Asian race, and the year of diagnosis (2013 to 2017) had time-varying associations with cancer mortality. Furthermore, a constant effect $\hat{\alpha } = -0.28$ was associated with the interaction term between the age group under 50 and the year of diagnosis group ranging from 2013 to 2017. A plot showing how the variables were selected during the algorithm is shown in Fig. S1 in the Online Appendix. Of note is that most of the iterations in the algorithm do not select a new term to include, but rather update one of the parameters or group of parameters that are already in the model. The plot shows that the year of diagnosis range 2013–2017 was selected first by the algorithm and frequently updated, supporting the fact that survival outcomes for patients with metastatic melanoma have improved in recent years.

**Fig. 3:**
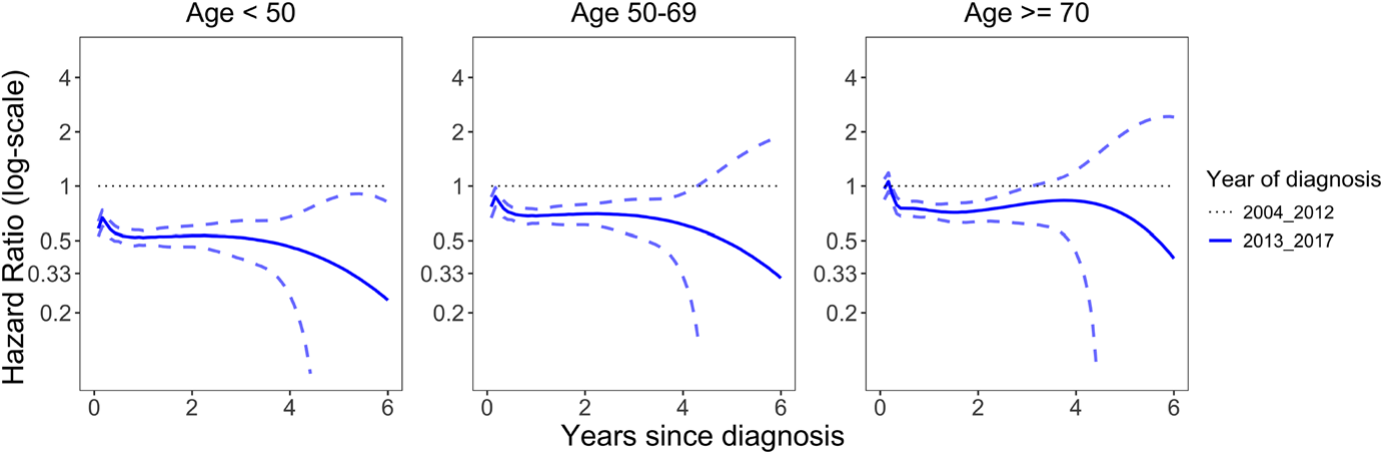
Estimated hazard ratios for melanoma cancer patients by age and diagnosis period. This figure presents the estimated hazard ratios (solid lines) and their corresponding $95\%$ confidence intervals (dashed lines). The figure comprises three subplots, each corresponding to a specific age group: under 50, 50–69, and over 70. Within each subplot, estimated hazard ratio curves are presented for non-Asian patients diagnosed in two distinct time periods: 2004–2012 and 2013–2017. The period of 2004 to 2012 serves as the reference group and is depicted with dotted lines. Each line within a subplot represents the hazard ratios for a specific diagnosis period, allowing for a comparative analysis of hazard ratios across various age groups and diagnosis years

Figure 3 presents the estimated hazard ratio curves for non-Asian patients, calculated using Eq. 2. The estimates are obtained from maximizing the penalized likelihood using P-splines. The $95\%$ confidence intervals were computed using standard errors derived via the delta method, with the variance estimated from the inverse of the information matrix (i.e. the negative second-order derivative of the penalized log likelihood), as suggested by (Luo et al. 2023). For patients diagnosed between 2013 and 2017 compared to those diagnosed between 2004 and 2012, there is a noticeable reduction in the hazard ratio for individuals under the age of 50, suggesting the recent benefit is stronger in the youngest age group.

## Discussion

This study introduces a novel boosting procedure for discrete failure time models that addresses key challenges in selecting important covariates and their interactions in right-censored data. The proposed method efficiently identifies both time-varying and time-independent effects, while preserving interpretability.

Our focus in this paper is on the algorithm to select variables and interactions in the presence of time-varying effects in a discrete time hazard model for large datasets. We do not consider statistical inference, other than presenting confidence intervals in the melanoma example. The confidence intervals we present are conditional on the selected model, and do not take into account the variable selection process. Developing methods for post-selection inference following a componentwise gradient boosting algorithm is a challenging task, worthy of further research.

Recent advances have extended tree-based machine learning methods, such as classification and regression trees (Berger 2018; Berger et al. 2019; Puth et al. 2020; Spuck et al. 2023) and random forests (Janitza 2015; Schmid et al. 2020), to the context of survival analysis, particularly in discrete-time frameworks. Some of these approaches can implicitly capture time-varying effects through recursive partitioning and interactions between time and covariates, without the need for strong parametric assumptions. These approaches are also useful at capturing interactions between covariates, including high order interactions. This differs from our approach in which we only consider 2-way interactions for reasons of parsimony and interpretability. While tree-based models can offer high predictive performance and flexibility, they often lack interpretability and make it challenging to isolate and characterize the functional form of time-varying covariate effects and to interpret interactions.

The SEER registry provides detailed cause-of-death information, allowing us to distinguish between cancer-related mortality and deaths from other causes. Thus this is a competing risks setting. In such settings it is possible to develop discrete time joint models for the multiple failure types. In our analysis, since death due to cancer is the primary outcome of interest, we adopt an alternative strategy by fitting a discrete-time cause-specific hazard models focused specifically on death due to cancer. This approach, which uses a “collapsed” versions of the log-likelihood and employs a “one-vs-all” model, has been previously explored in the literature (Lee et al. 2018; Schmid and Berger 2021). As shown by Lee et al. (2018), this modeling strategy yields consistent estimates of the cause-specific hazard function, making it a practical and theoretically sound choice for our discrete failure time setting.

A challenge with boosting approaches is when to stop the algorithm. Approaches that use BIC need a way of calculating the degrees of freedom. The method suggested in the literature for the boosting algorithm is not computationally feasible in our setting, so instead we adapted concepts of degrees of freedom from the smoothing literature, that require the calculation of Hessian matrices. The boosting algorithm is effective at selecting parameters to make non-zero, but we observed that the actual estimates of the parameters were noticeably biased towards zero. Re-fitting the selected model using likelihood based methods reduced the bias of the estimates, and the variability of the estimates was further controlled by maximizing a penalized likelihood, instead of the original likelihood. The above observations motivated our suggested way of deciding when to stop the boosting algorithm based on parameter re-estimation BIC. We found promising results using this approach, and it is computationally feasible, because the number of parameters in the selected model is not too large.

Imposing strong and weak hierarchical principles is very natural and easy to implement in the coordinate-wise boosting algorithm. While we considered hierarchy concerned with interactions and main effects, the concept could also be applied to not selecting time-varying coefficients before time-independent coefficients are selected. This approach is computationally feasible for large studies while maintaining accuracy. Simulations and applications using SEER data demonstrate the method’s effectiveness and flexibility in handling complex data structures. This work enhances researcher’s ability to fit flexible interpretable models to large datasets with many covariates thus providing a valuable tool for future large-scale studies.

Although boosting has demonstrated strong empirical performance and has been widely used for prediction and variable screening, comprehensive theoretical guarantees for variable selection and stopping rules in high-dimensional survival models remain limited, and rigorous asymptotic theory is still an active area of research (Bühlmann and Yu 2006). In particular, our proposed procedure involves component-wise updates, hierarchical interaction selection, and data-driven early stopping, all of which complicate a full theoretical treatment in the presence of right censoring and time-varying effects. While the proposed algorithm is motivated by well-established connections between boosting, regularization, and forward stagewise fitting, we do not establish formal results such as model selection consistency or asymptotic convergence rates in this work. Instead, we focus on methodological development and computational feasibility, and evaluate performance through extensive simulations and a large-scale registry application. Developing a rigorous asymptotic framework for boosting-based selection procedures in discrete failure time models is an important direction for future research.

## Supplementary Information

Below is the link to the electronic supplementary material.

Supplementary file 1 (pdf 929 KB)
