# Supplementary material for: Gradient boosting-based discrete failure time model for selecting time-varying effects and interactions
Source: Lifetime Data Anal. 2026 May 13;32(2):31. doi: 10.1007/s10985-026-09710-4 (PMC13167897; doi:10.1007/s10985-026-09710-4)
Supplement: Supplementary file 1 — (pdf 929 KB) [file 10985_2026_9710_MOESM1_ESM.pdf]

Supporting Information

1 Methods

1.1 Time-Varying Effects for Discrete Survival Model: Group-LASSO Variable Selection.

Group LASSO can be used to perform variable selection (Yuan and Lin, 2006; Yan and Huang, 2012). To facilitate its application within a time-varying coefficient framework, we follow the notation in Section 2.1 of the main manuscript. The log-likelihood is given by:

$$\begin{aligned}\ell_{gr}(\boldsymbol{\phi},\boldsymbol{\theta},\boldsymbol{\gamma}) = \frac{1}{\mathcal{N}}\sum_{i=1}^{n}\sum_{s=1}^{T_i}\Bigg\{ & y_{is}\log\left[g\left(\gamma_s+\sum_{j=1}^{P}\phi_j z_{ij}+\sum_{j=1}^{P}\boldsymbol{\theta}_j^{\top}\mathbf{B}(t_s)z_{ij}\right)\right] \\ & +(1-y_{is})\log\left[1-g\left(\gamma_s+\sum_{j=1}^{P}\phi_j z_{ij}+\sum_{j=1}^{P}\boldsymbol{\theta}_j^{\top}\mathbf{B}(t_s)z_{ij}\right)\right]\Bigg\}.\end{aligned}$$

Our loss function is the negative log-likelihood. The group lasso estimator, is defined as the value that minimizes

$$Q(\boldsymbol{\phi},\boldsymbol{\theta},\boldsymbol{\gamma}) = -\ell_{gr}(\boldsymbol{\phi},\boldsymbol{\theta},\boldsymbol{\gamma})+\lambda_n\sum_{j=1}^{P}\{W_{j1}|\phi_j|+W_{j2}||\boldsymbol{\theta}_j||\}, \tag{1}$$

with weight assignments $W_{j1}=1$ and $W_{j2}=K-1$. This structured approach allows the use of Group Lasso Methods for our model.

1.2 Newton's Method for Discrete Survival Model

In this subsection, we detail the use of Newton's Method with penalization for parameter estimation in our discrete survival model.

The log-likelihood function, essential for our model's estimation, is reiterated from the main paper as follows:

$$\ell(\boldsymbol{\eta}) = \sum_{i=1}^{n}\sum_{s=1}^{T_i}\left[y_{is}(\gamma_s+f(t_s;\mathbf{Z}_i))-\log\left(1+\exp\left(\gamma_s+f(t_s;\mathbf{Z}_i)\right)\right)\right], \tag{2}$$

where $f(t_s;\mathbf{Z}_i)=\sum_{j=1}^{P}\phi_j z_{ij}+\sum_{j=1}^{P}\boldsymbol{\theta}_j^{\top}\mathbf{B}(t_s)z_{ij}+\sum_{j<j'}\alpha_{jj'}z_{ij}z_{ij'}$.

To implement Newton's Method, we derive the score function and Hessian matrix.

The components of the score vector $c = c(\gamma, \phi, \boldsymbol{\theta}, \alpha)$ are

$$\frac{\partial \ell}{\partial \gamma_s} = \sum_{i=1}^{n} \left(y_{is} - \frac{1}{1 + \exp(-\gamma_s - f(t_s; \mathbf{Z}_i))} \right), \tag{3}$$

$$\frac{\partial \ell}{\partial \phi_j} = \sum_{i=1}^{n} \sum_{s=1}^{T_i} \left(y_{is} z_{ij} - \frac{z_{ij}}{1 + \exp(-\gamma_s - f(t_s; \mathbf{Z}_i))} \right), \tag{4}$$

$$\frac{\partial \ell}{\partial \theta_{jk}} = \sum_{i=1}^{n} \sum_{s=1}^{T_i} \left(y_{is} z_{ij} B_k(t_s) - \frac{z_{ij} B_k(t_s)}{1 + \exp(-\gamma_s - f(t_s; \mathbf{Z}_i)))} \right), \tag{5}$$

$$\frac{\partial \ell}{\partial \alpha_{jj'}} = \sum_{i=1}^{n} \sum_{s=1}^{T_i} \left(y_{is} z_{ij} z_{ij'} - \frac{z_{ij} z_{ij'}}{1 + \exp(-\gamma_s - f(t_s; \mathbf{Z}_i))} \right), \tag{6}$$

where $1 \le j \le P$ and $1 \le k \le K$.

The second derivatives of ℓ can be written as

$$H = \begin{pmatrix} H_{11} & H_{12} & H_{13} & H_{14} \\ H_{21} & H_{22} & H_{23} & H_{24} \\ H_{31} & H_{32} & H_{33} & H_{34} \\ H_{41} & H_{42} & H_{43} & H_{44} \end{pmatrix} = \begin{pmatrix} -\frac{\partial^2 \ell}{\partial \gamma \partial \gamma} & -\frac{\partial^2 \ell}{\partial \gamma \partial \phi} & -\frac{\partial^2 \ell}{\partial \gamma \partial \boldsymbol{\theta}^\top} & -\frac{\partial^2 \ell}{\partial \gamma \partial \alpha} \\ -\frac{\partial^2 \ell}{\partial \phi \partial \gamma} & -\frac{\partial^2 \ell}{\partial \phi \partial \phi} & -\frac{\partial^2 \ell}{\partial \phi \partial \boldsymbol{\theta}^\top} & -\frac{\partial^2 \ell}{\partial \phi \partial \alpha} \\ -\frac{\partial^2 \ell}{\partial \boldsymbol{\theta} \partial \gamma} & -\frac{\partial^2 \ell}{\partial \boldsymbol{\theta} \partial \phi} & -\frac{\partial^2 \ell}{\partial \boldsymbol{\theta} \partial \boldsymbol{\theta}^\top} & -\frac{\partial^2 \ell}{\partial \boldsymbol{\theta} \partial \alpha} \\ -\frac{\partial^2 \ell}{\partial \alpha \partial \gamma} & -\frac{\partial^2 \ell}{\partial \alpha \partial \phi} & -\frac{\partial^2 \ell}{\partial \alpha \partial \boldsymbol{\theta}^\top} & -\frac{\partial^2 \ell}{\partial \alpha \partial \alpha} \end{pmatrix}, \tag{7}$$

where H_{11} is diagonal with the sth element equal to:

$$-\frac{\partial^2 \ell}{\partial \gamma_s^2} = \sum_{i=1}^{n} \frac{\exp(-\gamma_s - f(t_s; \mathbf{Z}_i))}{(1 + \exp(-\gamma_s - f(t_s; \mathbf{Z}_i)))^2}.$$

The columns of H_{21}, H_{31}, and H_{41} are as follows:

$$-\frac{\partial^2 \ell}{\partial \phi_j \partial \gamma_s} = \sum_{i=1}^{n} \frac{\exp(-\gamma_s - f(t_s; \mathbf{Z}_i))}{(1 + \exp(-\gamma_s - f(t_s; \mathbf{Z}_i)))^2} z_{ij},$$

$$-\frac{\partial^2 \ell}{\partial \theta_{jk} \partial \gamma_s} = \sum_{i=1}^{n} \frac{\exp(-\gamma_s - f(t_s; \mathbf{Z}_i))}{(1 + \exp(-\gamma_s - f(t_s; \mathbf{Z}_i)))^2} z_{ij} B_k(t_s),$$

$$-\frac{\partial^2 \ell}{\partial \alpha_{jj'} \partial \gamma_s} = \sum_{i=1}^{n} \frac{\exp(-\gamma_s - f(t_s; \mathbf{Z}_i))}{(1 + \exp(-\gamma_s - f(t_s; \mathbf{Z}_i)))^2} z_j z_{j'}.$$

H_{22} is diagonal with jth element equal to:

$$-\frac{\partial^2 \ell}{\partial \phi_j^2} = \sum_{i=1}^{n} \frac{\exp(-\gamma_s - f(t_s; \mathbf{Z}_i))}{(1 + \exp(-\gamma_s - f(t_s; \mathbf{Z}_i)))^2} z_{ij}^2.$$

The columns of H_{32} and H_{42} are as follows:

$$-\frac{\partial^2 \ell}{\partial \theta_{jk} \partial \phi_j} = \sum_{i=1}^{n} \frac{\exp(-\gamma_s - f(t_s; \mathbf{Z}_i))}{(1 + \exp(-\gamma_s - f(t_s; \mathbf{Z}_i)))^2} z_{ij}^2 B_k(t_s),$$

$$-\frac{\partial^2 \ell}{\partial \alpha_{jj'} \partial \phi_j} = \sum_{i=1}^{n} \frac{\exp(-\gamma_s - f(t_s; \mathbf{Z}_i))}{(1 + \exp(-\gamma_s - f(t_s; \mathbf{Z}_i)))^2} z_{ij}^2 z_{ij'}.$$

$$H_{33} = -\frac{\partial^2 \ell}{\partial \theta_{jk} \partial \theta_{j'k'}} = \sum_{i=1}^{n} \sum_{s=1}^{T_i} \frac{\exp(-\gamma_s - f(t_s; \mathbf{Z}_i))}{(1 + \exp(-\gamma_s - f(t_s; \mathbf{Z}_i)))^2} z_{ij} z_{ij'} B_k(s) B_{k'}(s),$$

and

$$H_{43} = -\frac{\partial^2 \ell}{\partial \theta_{jk} \partial \alpha_j} = \sum_{i=1}^{n} \sum_{s=1}^{T_i} \frac{\exp(-\gamma_s - f(t_s; \mathbf{Z}_i))}{(1 + \exp(-\gamma_s - f(t_s; \mathbf{Z}_i)))^2} z_{ij} B_k(t_s) z_{ij} z_{ij'}.$$

H_{44} is diagonal with each element equal to:

$$H_{44} = -\frac{\partial^2 \ell}{\partial \alpha_{jj'} \partial \alpha_{j''j'''}} = \sum_{i=1}^{n} \sum_{s=1}^{T_i} \frac{\exp(-\gamma_s - f(t_s; \mathbf{Z}_i))}{(1 + \exp(-\gamma_s - f(t_s; \mathbf{Z}_i)))^2} z_{ij} z_{ij'} z_{ij''} z_{ij'''}.$$

With the score function and Hessian matrix, we can implement the Newton's method. Note that we leverage diagonal matrix properties to avoid complicated matrix inversion, thus enhancing computational speed and reducing memory usage.

1.3 Calculation of Bias, SD and IMSE

In this section, we provide details of the calculations for the average bias, integrated mean squared error (IMSE), and average standard deviation (SD). We generated 100 data replicates using the notation $d = 1, \ldots, D$, with $D = 100$. Let $t_1, \ldots, t_S$ be the distinct failure times indexed by $s = 1, \ldots, S$. We calculate the estimated curves for each of the P covariates:

$$\widehat{\boldsymbol{\beta}}_{j_d}(t_s) = \widehat{\boldsymbol{\theta}}_{j_d}^T \boldsymbol{B}(t_s), \quad j = 1, \ldots, P, \quad d = 1, \ldots, D,$$

where $\widehat{\boldsymbol{\theta}}_{j_d}^T$ represents the estimated time-varying effect related coefficients obtained from the proposed methods in the d_{th} data replicate. Let $\widehat{\boldsymbol{\gamma}}_{s_d}$ represent the estimated baseline hazard for d_{th} data replicate at time t_s. Let $\widehat{\boldsymbol{\phi}}_{j_d}, \widehat{\boldsymbol{\alpha}}_{jj'_d}$ represent the estimated time-independent effect and coefficients of the interaction terms for from the d_{th} data replicate. Note $1 \leq j < j' \leq P$. We calculate these metrics for all baseline hazard, time-varying and time-independent effects, and interaction terms in our setting. For the baseline hazard, the metrics are calculated as:

$$\text{bias} = \frac{1}{DS} \sum_{d=1}^{D} \sum_{s=1}^{S} \left(|\widehat{\boldsymbol{\gamma}}_{s_d} - \boldsymbol{\gamma}_{s_d}| \right),$$

$$\text{SD} = \frac{1}{S} \sum_{s=1}^{S} \sqrt{\frac{\sum_{d=1}^{D} \left(\widehat{\boldsymbol{\gamma}}_{s_d} - \frac{1}{D} \sum_{d=1}^{D} \widehat{\boldsymbol{\gamma}}_{s_d} \right)^2}{D}},$$

$$\text{IMSE} = \frac{1}{DS} \sum_{d=1}^{D} \sum_{s=1}^{S} \left(\widehat{\boldsymbol{\gamma}}_{s_d} - \boldsymbol{\gamma}_{s_d} \right)^2.$$

For the time-varying and time-independent effects, the metrics are calculated as:

$$\text{bias} = \frac{1}{DPS}\sum_{d=1}^{D}\sum_{j=1}^{P}\sum_{s=1}^{S}\left(\left|\widehat{\boldsymbol{\beta}}_{j_d}(t_s)+\widehat{\boldsymbol{\phi}}_{j_d}-\boldsymbol{\beta}_{j_d}(t_s)-\boldsymbol{\phi}_{j_d}\right|\right),$$

$$\text{SD} = \frac{1}{PS}\sum_{j=1}^{P}\sum_{s=1}^{S}\sqrt{\frac{\sum_{d=1}^{D}\left(\widehat{\boldsymbol{\beta}}_{j_d}(t_s)+\widehat{\boldsymbol{\phi}}_{j_d}-\frac{1}{D}\sum_{d=1}^{D}\widehat{\boldsymbol{\beta}}_{j_d}(t_s)-\frac{1}{D}\sum_{d=1}^{D}\widehat{\boldsymbol{\phi}}_{j_d}\right)^2}{D}},$$

$$\text{IMSE} = \frac{1}{DPS}\sum_{d=1}^{D}\sum_{j=1}^{P}\sum_{s=1}^{S}\left(\widehat{\boldsymbol{\beta}}_{j_d}(t_s)+\widehat{\boldsymbol{\phi}}_{j_d}-\boldsymbol{\beta}_{j_d}(t_s)-\boldsymbol{\phi}_{j_d}\right)^2.$$

For the interaction terms, the metrics are calculated as:

$$\text{bias} = \frac{2}{DSP(P-1)}\sum_{d=1}^{D}\sum_{s=1}^{S}\sum_{j<j'}\left(\left|\widehat{\boldsymbol{\alpha}}_{jj'_d}-\boldsymbol{\alpha}_{jj'_d}\right|\right),$$

$$\text{SD} = \frac{2}{SP(P-1)}\sum_{s=1}^{S}\sum_{j<j'}\sqrt{\frac{\sum_{d=1}^{D}\left(\widehat{\boldsymbol{\alpha}}_{jj'_d}-\frac{1}{D}\sum_{d=1}^{D}\boldsymbol{\alpha}_{jj'_d}\right)^2}{D}},$$

$$\text{IMSE} = \frac{2}{DSP(P-1)}\sum_{d=1}^{D}\sum_{s=1}^{S}\sum_{j<j'}\left(\widehat{\boldsymbol{\alpha}}_{jj'_d}-\boldsymbol{\alpha}_{jj'_d}\right)^2.$$

2 Connections between DiscBoosting and Steepest Ascent Methods

We now show an implicit connection between the proposed DiscBoosting algorithm and the steepest ascent algorithm.

2.1 Review of Steepest Ascent Methods

In this section we do a brief review of the existing steepest ascent methods (Boyd et al., 2004). We consider methods maximizing a log-likelihood:

$$\max_{\boldsymbol{\beta}} \ell(\boldsymbol{\beta}).$$

We utilize a first-order condition,

$$\ell(\widehat{\boldsymbol{\beta}}+\alpha\boldsymbol{\mu}) \leq \ell(\widehat{\boldsymbol{\beta}})+\alpha\nabla\ell(\widehat{\boldsymbol{\beta}})^T\boldsymbol{\mu},$$

where $\widehat{\boldsymbol{\beta}}$ is the current estimate; $\boldsymbol{\mu}$ is the update direction of $\boldsymbol{\beta}$; α is a small positive value, and the term $\nabla\ell(\widehat{\boldsymbol{\beta}})^T\boldsymbol{\mu}$ is the directional derivative along $\boldsymbol{\mu}$. To increase $\ell(\boldsymbol{\beta})$, We aim to find an ascent direction, $\boldsymbol{\mu}$ such that $\nabla\ell(\widehat{\boldsymbol{\beta}})^T\boldsymbol{\mu} > 0$. Moreover, to improve the optimization efficiency, we identify an update

direction, along which $\ell(\boldsymbol{\beta})$ ascends most rapidly. This motivates a steepest ascent update direction,

$$\boldsymbol{\mu}^{\star} = \underset{\boldsymbol{\mu}}{\operatorname{argmax}}\{\nabla\ell(\widehat{\boldsymbol{\beta}})^{T}\boldsymbol{\mu} \mid ||\boldsymbol{\mu}||_{\dagger} = 1\},$$

where $||\cdot||_{\dagger}$ is a vector norm on $\mathbb{R}^p$, and $\boldsymbol{\mu}^{\star}$ is the direction of unit norm (termed as the normalized steepest ascent direction) that gives the largest increase in the linear approximation of ℓ,

$$\nabla\ell(\widehat{\boldsymbol{\beta}})^{\top}\boldsymbol{\mu}^{\star} = \max\{\nabla\ell(\widehat{\boldsymbol{\beta}})^{\top}\boldsymbol{\mu} \mid \|\boldsymbol{\mu}\|_{\dagger} = 1\} = \|\nabla\ell(\widehat{\boldsymbol{\beta}})\|_{\star},$$

where $\|\cdot\|_{\star}$ is the dual norm of $\|\cdot\|_{\dagger}$.

Because the directional derivative $\nabla\ell(\widehat{\boldsymbol{\beta}})^{\top}\boldsymbol{\mu}$ can be arbitrarily large as the magnitude of $\boldsymbol{\mu}$ increases, the constraint that $\boldsymbol{\mu}$ is of a unit norm enables fair comparisons among directions.

2.2 Achieving Variable Selection Via Steepest Ascent Methods

The selection of a norm, denoted as $|\cdot|_{\dagger}$, is important for enhancing computational efficiency and ensuring numerical stability in different ascent methodologies. To achieve variable selection, we select a norm which updates one single group of parameters at each iteration.

To accomplish group variable selection, we adopt the ℓ_1/quadratic norm, which is elaborated in Definition 1.

Definition 1 (ℓ_1/quadratic norm) Suppose $\boldsymbol{\beta}$ can be divided into G groups $\boldsymbol{\beta} = (\boldsymbol{\beta}_1^{\top}, \ldots, \boldsymbol{\beta}_G)^{\top}$, where each $\boldsymbol{\beta}_j$ is a q_j-dimensional vector, $j = 1, \ldots, G$. For ease of notation and without loss of generality, we assume $q_j = q$ for all groups j. The ℓ_1/quadratic norm associated with a set of $q \times q$ dimensional positive definite matrices $\mathbf{H}_1, \ldots, \mathbf{H}_p$, is defined as

$$\|\boldsymbol{\beta}\|_{\ell_1/\text{quadratic}} = \sum_{j=1}^{p} \|\boldsymbol{\beta}_j\|_{\mathbf{H}_j} = \sum_{j=1}^{p} \|\mathbf{H}_j^{1/2}\boldsymbol{\beta}\|_2 = \sum_{j=1}^{p} \left(\boldsymbol{\beta}_j^{\top}\mathbf{H}_j\boldsymbol{\beta}_j\right)^{1/2},$$

where the quadratic norm is applied within each group, and the ℓ_1 norm is applied across groups Boyd et al. (2004).

Utilizing this norm as a foundation, we subsequently introduce the Block-Wise Modified Steepest Ascent Algorithm. The detailed algorithm is given below.

Given a current estimate $\widehat{\boldsymbol{\beta}}$, the normalized steepest ascent direction for ℓ_1/quadratic norm is given by a block-wise update

$$\boldsymbol{\mu}^{\star} = \underset{\boldsymbol{\mu}}{\operatorname{argmax}}\left\{\nabla\ell(\widehat{\boldsymbol{\beta}})^{\top}\boldsymbol{\mu} \;\middle|\; \sum_{j=1}^{p} ||\boldsymbol{\mu}_j||_{\mathbf{H}_j} = 1\right\} = (0, \ldots, 0, \boldsymbol{\mu}_{j^*}^{\top}, 0, \ldots, 0)^{\top}, \quad (8)$$

where $\boldsymbol{\mu}_j$ is a k-dimensional vector corresponding to the j-th block of $\boldsymbol{\mu}$,

$$j^\star = \underset{j}{\operatorname{argmax}} \left(||\nabla \ell(\widehat{\boldsymbol{\beta}})_j||_{\mathbf{H}_j^{-1}} \right),$$

and $\boldsymbol{\mu}_{j^\star}$ is given by

$$\boldsymbol{\mu}_{j^\star} = \left(\nabla \ell(\widehat{\boldsymbol{\beta}})_{j^\star}^\top \{\mathbf{H}_{j^\star}\} \nabla \ell(\widehat{\boldsymbol{\beta}})_{j^\star} \right)^{-1/2} \{\mathbf{H}_{j^\star}\}^{-1} \nabla \ell(\widehat{\boldsymbol{\beta}})_{j^\star}. \tag{9}$$

The corresponding unnormalized steepest ascent direction is given by

$$\widetilde{\boldsymbol{\mu}} = \max_j \left\| \nabla \ell(\hat{\boldsymbol{\beta}}_j) \right\|_{\mathbf{H}_j^{-1}} \boldsymbol{\mu}^* = (0, \ldots, 0, \widetilde{\boldsymbol{\mu}}_{j*}^T, 0, \ldots, 0)^T, \tag{10}$$

where $\widetilde{\boldsymbol{\mu}}_{j*} = \mathbf{H}_{j*}^{-1} \nabla \ell(\widehat{\boldsymbol{\beta}})_{j*}$. This leads to the Modified Block-Wise Steepest Ascent Algorithm that iteratively pursues the optimal block-wise direction maximizing the directional derivative.

Algorithm 1 Modified Block-Wise Steepest Ascent Algorithm

(1) Initialize $\widehat{\boldsymbol{\beta}}^{(0)} = \mathbf{0}$.
(2) For $m = 1, 2, \ldots, M_{stop}$, identify $j_m^\star$ as

$$j_m^\star = \underset{j}{\operatorname{argmax}} \left(||\nabla \ell(\widehat{\boldsymbol{\beta}}^{(m-1)})_j||_{\mathbf{H}_j^{-1}} \right)$$

and update $\boldsymbol{\beta}$ by

$$\widehat{\boldsymbol{\beta}}_j^{(m)} = \widehat{\boldsymbol{\beta}}_j^{(m-1)} + \nu \, \widetilde{\boldsymbol{\mu}}_{j^\star}^{(m)} \quad \text{if} \quad j = j_m^\star,$$
$$\widehat{\boldsymbol{\beta}}_j^{(m)} = \widehat{\boldsymbol{\beta}}_j^{(m-1)} \quad \text{if} \quad j \neq j_m^\star,$$

where $\widetilde{\boldsymbol{\mu}}_{j^\star}^{(m)}$ is computed as

$$\widetilde{\boldsymbol{\mu}}_{j^\star}^{(m)} = \mathbf{H}_{j*}^{-1} \nabla \ell(\widehat{\boldsymbol{\beta}}^{(m-1)})_{j*},$$

and ν is a small positive value (e.g. 0.01) controlling the learning rate.
(3) Repeat iterations until $m = M_{stop}$.

Note that variable selection can be achieved if the procedure is set to stop at a finite number of steps since only one group of variables is updated during each iteration. Effectively, the step number M_{stop} is a tuning parameter and can be determined by the proposed stopping criteria in the main paper.

2.3 DiscBoosting

In this section, we present the link between the proposed DiscBoosting approach and the steepest ascent method to show that the updates made by

DiscBoosting effectively maximize the surrogate function of our objective function, within certain constraints. This procedure is equivalent to employing the steepest ascent algorithm as outlined in Algorithm 1.

For clarity, we adopt a notation slightly divergent from that used in the main text, focusing exclusively on the representation of the algorithm within the context of group variable selection. Following the simplified setting, the proposed DiscBoosting can be written as below:

Algorithm 2 DiscBoosting Algorithm for Group Variable Selection

Initialize $\widehat{\boldsymbol{\theta}}^{(0)} = \mathbf{0}$. For $m = 1, 2, \ldots, M_{stop}$, iterate the following:

(1) Given $\widehat{\boldsymbol{\beta}}^{(m-1)}$, compute pseudo-outcomes $\mathbf{U}^{(m)} = (U_1^{(m)}, \ldots, U_n^{(m)})$, where

$$U_i^{(m)} = \frac{\partial \ell(\boldsymbol{\eta})}{\partial \eta_i}\Big|_{\eta_i = \hat{\eta}_i^{(m-1)}},\ i = 1, \ldots, n,$$

where $\boldsymbol{\eta} = (\eta_1, \ldots, \eta_n)^\top$, with $\eta_i = \mathbf{Z}_i^\top \boldsymbol{\beta}$ for $i = 1, \ldots, n$.

(2) Fit the pseudo-outcomes to each group of covariates by an univariate least squares and identify group $j_m^\star$ by

$$j_m^\star = \underset{j}{\operatorname{argmin}} \left(\mathbf{U}^{(m)} - \mathbf{Z}_j \widetilde{\boldsymbol{\beta}}_j\right)^\top \left(\mathbf{U}^{(m)} - \mathbf{Z}_j \widetilde{\boldsymbol{\beta}}_j\right), \tag{11}$$

where $$\mathbf{Z}_j = (\mathbf{Z}_{1j}^\top, \ldots, \mathbf{Z}_{nj}^\top)^\top,\ \text{and}\ \widetilde{\boldsymbol{\beta}}_j = (\mathbf{Z}_j^\top \mathbf{Z}_j)^{-1} \mathbf{Z}_j^\top \mathbf{U}^{(m)} \tag{12}$$

(3) Update $\boldsymbol{\beta}$ by

$$\widehat{\boldsymbol{\beta}}_j^{(m)} = \widehat{\boldsymbol{\beta}}_j^{(m-1)} + \nu\, \widetilde{\boldsymbol{\beta}}_j \ \text{ if } \ j = j_m^\star,$$
$$\widehat{\boldsymbol{\beta}}_j^{(m)} = \widehat{\boldsymbol{\beta}}_j^{(m-1)} \text{ if } j \neq j_m^\star.$$

With the DiscBoosting and Modified Block-Wise Steepest Ascent Algorithm summarized above, we now show the connection between the two algorithms.

Suppose we set the $\mathbf{H}_j = \mathbf{Z}_j^\top \mathbf{Z}_j$, where $\mathbf{Z}_j$ is a $n \times q$-dimensional covariate matrix corresponding to the j-th group of predictors. By the chain rule of differentiation,

$$\nabla \ell(\widehat{\boldsymbol{\beta}}^{(m-1)})_j = \sum_{i=1}^{n} \frac{\partial \ell(\boldsymbol{\eta})}{\partial \eta_i} \frac{\partial \eta_i}{\boldsymbol{\beta}_j}\Big|_{\boldsymbol{\beta} = \widehat{\boldsymbol{\beta}}^{(m-1)}} = \mathbf{Z}_j^\top \mathbf{U}^{(m)},$$

where $\mathbf{U}^{(m)} = (U_1^{(m)}, \ldots, U_n^{(m)})$ is the vector of pseudo outcomes. It follows that

$$\begin{aligned}\underset{j}{\operatorname{argmax}} \left(||\nabla \ell(\widehat{\boldsymbol{\beta}}^{(m-1)})_j||_{(\mathbf{Z}_j^\top \mathbf{Z}_j)^{-1}}\right) &= \underset{j}{\operatorname{argmax}} \left\{(\mathbf{Z}_j^\top \mathbf{U}^{(m)})^\top (\mathbf{Z}_j^\top \mathbf{Z}_j)^{-1} \mathbf{Z}_j^\top \mathbf{U}^{(m)}\right\} \\ &= \underset{j}{\operatorname{argmin}} \left(\mathbf{U}^{(m)} - \mathbf{Z}_j \widetilde{\boldsymbol{\beta}}_j\right)^\top \left(\mathbf{U}^{(m)} - \mathbf{Z}_j \widetilde{\boldsymbol{\beta}}_j\right),\end{aligned}$$

where $\widetilde{\boldsymbol{\beta}}_j = (\mathbf{Z}_j^\top \mathbf{Z}_j)^{-1}\mathbf{Z}_j^\top \mathbf{U}$. Therefore, the Modified Block-wise Gradient Boosting is equivalent to DiscBoosting.

2.4 Degrees of Freedom

First we define $\widetilde{\mathbf{x}}_{\mathsf{g}_m} = (\mathbf{x}_{\mathsf{g}_m}^\top \otimes \mathbf{B}_1, \ldots, \mathbf{x}_{\mathsf{g}_m}^\top \otimes \mathbf{B}_K)^\top$. Note that $\widetilde{\mathbf{x}}_{\mathsf{g}_m}$ is a $N \times k$ dimensional covariate vector and $\mathbf{B}_k$ is the k-th column of the B-spline basis $(\mathbf{B}_k(t_1), \ldots, \mathbf{B}_k(t_S))$.

For iteration step m:

$$\mathcal{H}^{\hat{\mathsf{g}}_m} = \begin{cases} \mathbf{x}_{\mathsf{g}_m}(\mathbf{x}_{\mathsf{g}_m}^\top \mathbf{x}_{\mathsf{g}_m})^{-1}\mathbf{x}_{\mathsf{g}_m}^\top & \text{if } \mathsf{g}_m \in \mathsf{P}_{\mathrm{TI}} \text{ or } \mathsf{g}_m \in \mathsf{G}_2^{[m]}, \\ \widetilde{\mathbf{x}}_{\mathsf{g}_m}(\widetilde{\mathbf{x}}_{\mathsf{g}_m}^\top \widetilde{\mathbf{x}}_{\mathsf{g}_m})^{-1}\widetilde{\mathbf{x}}_{\mathsf{g}_m}^\top & \text{if } \mathsf{g}_m \in \mathsf{P}_{\mathrm{TV}}, \end{cases}$$

and

$$\hat{p}^{[m]} = \frac{\exp(\boldsymbol{\eta})}{1+\exp(\boldsymbol{\eta})},$$
$$W^{[m]} = \mathrm{diag}(\hat{p}^{[m]}(1-\hat{p}^{[m]})),$$

The stopping step is determined by calculating the BIC at each iteration, as expressed by the following equation:

$$\mathrm{BIC}(m) = -2\ell + \log(n)\mathrm{df}(m), \tag{13}$$

where the degrees of freedom is given by

$$\mathrm{df}(m) = \mathrm{trace}(\mathcal{B}_m), \tag{14}$$

and the algorithm stops at the value of m that minimizes $\mathrm{BIC}(m)$.

2.5 Step-by-Step DiscBoosting Procedure

To provide a clear and structured overview of the proposed methodology, we summarize the step-by-step Discrete Failure Time Boosting (DiscBoosting) procedure in Algorithm 3. This formalizes the iterative forward-selection approach described in Section 2.3 of the main manuscript.

Algorithm 3 Discrete Failure Time Boosting (DiscBoosting) Procedure

Initialization:
Set step sizes $\nu = 0.5$ and $\nu_\gamma = 1$.
Initialize parameters: $\boldsymbol{\gamma}^{[0]} = \mathbf{0}$, $\boldsymbol{\phi}^{[0]} = \mathbf{0}$, $\boldsymbol{\theta}^{[0]} = \mathbf{0}$, and $\boldsymbol{\alpha}^{[0]} = \mathbf{0}$.
Compute initial gradient $\mathbf{U}^{[0]} = \mathbf{U}(\boldsymbol{\gamma}^{[0]}, \boldsymbol{\phi}^{[0]}, \boldsymbol{\theta}^{[0]}, \boldsymbol{\alpha}^{[0]})$.
Initialize candidate sets: $\mathsf{P}_{\mathrm{TI}} = \{1, \ldots, P\}$, $\mathsf{P}_{\mathrm{TV}} = \{P+1, \ldots, 2P\}$, and $\mathsf{G}_2^{[0]} = \emptyset$.
Set iteration counter $m = 0$.
repeat
  Increase iteration: $m \leftarrow m + 1$.
  Update Gradient: Compute current gradient vector $\mathbf{U}^{[m-1]}$ evaluated at $\boldsymbol{\eta}^{[m-1]}$.
  Update Baseline Hazard:
    Calculate least squares estimate: $\widetilde{\boldsymbol{\gamma}}^{[m]} = \mathrm{argmin}_{\boldsymbol{\gamma}} \sum_{i=1}^{n} \sum_{s=1}^{T_i} (U_{is}^{[m-1]} - \gamma_s)^2$
    Update baseline hazard: $\widehat{\boldsymbol{\gamma}}^{[m]} = \widehat{\boldsymbol{\gamma}}^{[m-1]} + \nu_\gamma \widetilde{\boldsymbol{\gamma}}^{[m]}$
    Update gradient: $U_{is}^{[m-1]} \leftarrow U_{is}^{[m-1]} - \widetilde{\gamma}_s^{[m]}$
  Select Best Parameter ($\hat{\mathfrak{g}}_m$):
    Select the single term $\hat{\mathfrak{g}}_m \in \mathsf{P}_{\mathrm{TI}} \cup \mathsf{P}_{\mathrm{TV}} \cup \mathsf{G}_2^{[m-1]}$ that minimizes the least squares loss against the updated gradient.
    Calculate the corresponding estimate ($\widetilde{\phi}_{\hat{\mathfrak{g}}_m}$, $\widetilde{\boldsymbol{\theta}}_{\hat{\mathfrak{g}}_m}$, or $\widetilde{\alpha}_{\hat{\mathfrak{g}}_m}$).
  Apply Update:
  if $\hat{\mathfrak{g}}_m \in \mathsf{P}_{\mathrm{TI}}$ (Time-Independent) **then**
    $\hat{\phi}_{\hat{\mathfrak{g}}_m}^{[m]} = \hat{\phi}_{\hat{\mathfrak{g}}_m}^{[m-1]} + \nu \widetilde{\phi}_{\hat{\mathfrak{g}}_m}$
  else if $\hat{\mathfrak{g}}_m \in \mathsf{P}_{\mathrm{TV}}$ (Time-Varying) **then**
    $\hat{\boldsymbol{\theta}}_{\hat{\mathfrak{g}}_m}^{[m]} = \hat{\boldsymbol{\theta}}_{\hat{\mathfrak{g}}_m}^{[m-1]} + \nu \widetilde{\boldsymbol{\theta}}_{\hat{\mathfrak{g}}_m}$
  else if $\hat{\mathfrak{g}}_m \in \mathsf{G}_2^{[m-1]}$ (Interaction) **then**
    $\hat{\alpha}_{\hat{\mathfrak{g}}_m}^{[m]} = \hat{\alpha}_{\hat{\mathfrak{g}}_m}^{[m-1]} + \nu \widetilde{\alpha}_{\hat{\mathfrak{g}}_m}$
  end if
  Update Candidate Sets:
    Update active main effects $\mathsf{G}_1^{[m]}$ by adding the newly selected effect (if any).
    Update candidate interactions $\mathsf{G}_2^{[m]} = \{(j, j') : j, j' \in \mathsf{G}_1^{[m]}\}$.
until $m = m_{stop}$ ▷ Determined by Boosting BIC or Parameter Re-estimation BIC

3 Additional Simulation Studies

3.1 High Dimensional Variable Selection

To assess the performance of the proposed DiscBoosting method under higher signal densities, we conducted additional simulation studies with $P = 100$ covariates. The data generation process followed the setup described in Section 3.1 of the main manuscript regarding the generation of continuous covariates $\boldsymbol{Z}$ and failure times, but with an increased number of non-zero effects. We evaluated two scenarios: a moderate setting with $n = 1000$ and 20 true signals (10 time-varying, 10 time-independent) and a large setting with $n = 2000$ and 50 true signals (20 time-varying, 30 time-independent).

The non-zero coefficients were assigned as follows: the time-varying coefficients $\beta_j(t)$ were generated by cycling through three functions: $\cos(\pi t/50)$, $\sin(3\pi t/80)$, and $-1+\exp(-0.25t)$. The time-independent coefficients were set by alternating values of 1 and -1.

Table S1 summarizes the results based on 100 replications. In the large signal setting (50 signals), DiscBoosting maintained high performance (SE = 0.98, SP = 0.95), while Group LASSO's specificity dropped to nearly zero (0.03), essentially selecting almost all candidate variables as informative. Notably, in identifying time-varying (TV) effects specifically, DiscBoosting consistently demonstrated superior sensitivity and tighter control over false positives compared to Group LASSO across both settings. These results demonstrate that the proposed DiscBoosting method provides a much more favorable and parsimonious balance between sensitivity and specificity, accurately identifying true predictors even as the signal-to-noise ratio increases.

Table S1: Simulation results for moderate (20) and large (50) numbers of informative signals ($P = 100$). The sample size is $n = 1000$ for the moderate setting and $n = 2000$ for the large setting. False positive (FP) is the number of predictors selected by the algorithms when the true effect was zero. True positive (TP) is the number of predictors correctly identified. False negative (FN) is the number of true signals that the algorithms did not select. True negative (TN) is the number of noises correctly identified as having no effect. Sensitivity (SE) is calculated as the number of correctly chosen signals divided by the total number of true signals. Specificity (SP) is the number of correctly ignored noises divided by the total number of noises. Positive predictive value (PPV) is TP divided by the total number of selected predictors. Negative predictive value (NPV) is TN divided by the total number of ignored predictors.

Setting	Measure	Method	FP (sd)	FN (sd)	SE (sd)	SP (sd)	PPV (sd)	NPV (sd)
Moderate number of signals (20)	TI or TV	Boosting	11.26 (3.98)	2.67 (2.20)	0.87 (0.11)	0.86 (0.05)	0.62 (0.08)	0.04 (0.03)
		Group Lasso	44.65 (7.73)	0.00 (0.00)	1.00 (0.00)	0.44 (0.10)	0.31 (0.04)	0.00 (0.00)
	TV	Boosting	17.24 (4.82)	3.73 (1.66)	0.63 (0.17)	0.81 (0.05)	0.27 (0.08)	0.05 (0.02)
		Group Lasso	33.58 (7.65)	4.01 (1.57)	0.60 (0.16)	0.63 (0.08)	0.15 (0.04)	0.07 (0.02)
Large number of signals (50)	TI or TV	Boosting	2.62 (1.57)	1.00 (0.80)	0.98 (0.02)	0.95 (0.03)	0.95 (0.03)	0.02 (0.02)
		Group Lasso	48.30 (1.63)	0.00 (0.00)	1.00 (0.00)	0.03 (0.03)	0.51 (0.01)	0.00 (0.00)
	TV	Boosting	18.82 (2.94)	4.95 (1.60)	0.75 (0.08)	0.76 (0.04)	0.45 (0.05)	0.07 (0.02)
		Group Lasso	73.43 (3.44)	0.22 (0.44)	0.99 (0.02)	0.08 (0.04)	0.21 (0.01)	0.03 (0.07)

3.2 Overall Evaluation of Variable Selection in High-Dimensional Settings

To comprehensively evaluate the DiscBoosting algorithm's ability to simultaneously identify main effects, time-varying effects, and interaction terms, we conducted an extended simulation study. The data generation process followed the underlying structure described in Section 3.2 of the main manuscript, but we scaled the dimensions to represent a more challenging, high-dimensional setting. We increased the sample size to $n = 2000$ and expanded the number of main covariates to 40. This generated 780 possible two-way interaction terms, yielding a total of 820 candidate predictors. In this scenario, all 40 main co-

variates were established as true signals: 20 were assigned time-varying effects, and 20 were assigned time-independent constant effects (alternating between -1 and 1). The time-varying coefficients were defined using periodic and exponential curves, specifically: $1+\cos(\pi t/50)$, $-1+\exp(-0.25t)$, $1-\cos(\pi t/30)$, $\sin(3\pi t/80)$, and $-\sin(\pi t/30)$. Among the 780 interaction terms, 6 pairs were assigned non-zero effects.

The selection performance of the algorithm was evaluated under strong, weak, and no hierarchy constraints, as summarized in Table S2. The results indicate that the DiscBoosting procedure successfully scales to handle larger dimensional spaces while reliably identifying all three effect types. As expected, a strong hierarchy yields a highly conservative model with high specificity for interaction terms, though at a slight cost to sensitivity. Conversely, relaxing the hierarchy constraints allows the algorithm to capture nearly all true interaction signals while maintaining an exceptionally low false positive rate. Note that because all 40 main effects were generated as true signals, there are no noise variables among the main covariates. Consequently, FP are identical for both the 'Overall' and 'Interactions' metrics.

In addition to variable selection, we evaluated the parameter estimation accuracy after the selection procedure. Table S3 presents the absolute bias, average standard deviation (SD), and integrated mean squared error (IMSE) across the different scenarios. Discrete failure time models were fitted employing Newton's method using P-spline and Smoothing-spline penalties. Consistent with the findings in Section 3.2 of the main manuscript, the Full model, which lacks variable selection, consistently exhibits the highest bias, SD, and IMSE. The combination of gradient boosting coordinate-wise variable selection, penalized likelihood model fitting, and parameter re-estimation BIC successfully reduces the bias inherent in raw boosting estimates towards zero and significantly reduces variability. These results confirm that the DiscBoosting framework maintains high estimation accuracy in high-dimensional settings, particularly when hierarchy constraints are appropriately tuned.

3.3 Computational Efficiency and Scalability

To assess the computational feasibility of the proposed DiscBoosting algorithm, we conducted a simulation study examining the relationship between sample size, covariate dimensionality, and computation time. The data generation process followed the identical setting described in Section 3.1 of the main manuscript for high-dimensional variable selection, where we compared the runtime of DiscBoosting (using the Boosting BIC stopping criterion) against Group LASSO using 5-fold cross-validation.

As illustrated in Figure S1, the DiscBoosting method is consistently faster than Group LASSO (5-fold CV) and maintains a highly predictable, linear scalability with respect to both sample size N and the number of covariates P. Specifically, Group LASSO encountered convergence issues, particularly at $N = 400$ and $P = 500$, leading to unstable runtimes and numerical failures.

These results confirm that DiscBoosting provides a more efficient and reliable alternative for large-scale survival analysis where standard penalized regression often struggles with stability.

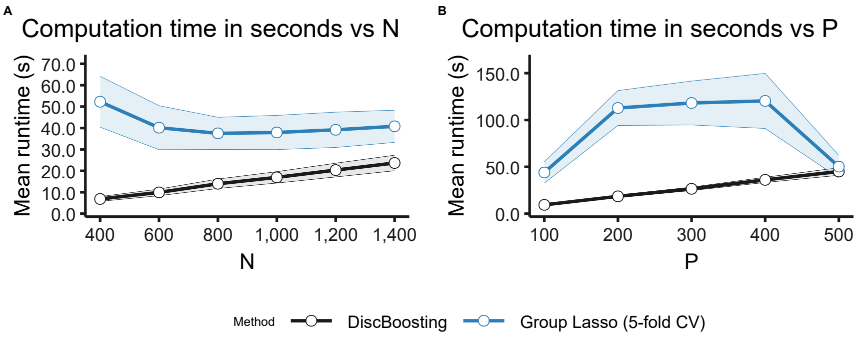

Fig. S1: Average computation time (in seconds) of the proposed DiscBoosting algorithm. Panel (a) illustrates the relationship between runtime and sample size, with N varying from 400 to 1400 and fixed dimension $P = 100$. Panel (b) illustrates the relationship between runtime and the number of covariates, with P varying from 100 to 500 and fixed sample size $N = 500$. The linear trends observed in both panels demonstrate the algorithm's scalability and efficiency.

3.4 Simulation Based on Fitted Results from Melanoma Cancer

In our investigation into metastatic melanoma cancer, we applied the DiscBoosting procedure to identify important covariates. Through our method, we selected the following variables: age groups under 50 and over 70, Asian race, and diagnosis years of 2013 and 2017 and identify them as having time-varying effects. Furthermore, the interaction between the age group under 50 and the year of diagnosis group ranging from 2013 to 2017 is selected.

To generate realistic simulation data that mimics the melanoma data, we employed Newton's method with P-spline on the metastatic melanoma dataset to obtain parameter estimates. We included main effects (time-varying) for all age groups, race groups, and diagnosis years, along with an interaction effect (time-independent) between the age group under 50 and diagnosis years from 2013 to 2017. The simulation setting was conducted with a sample size of $n = 10173$ and $P = 8$ predictors. The total number of candidate terms (including all possible two-way interaction terms) was 36. The predictors $\mathbf{Z}$ were the same as for the Melanoma cancer data. Failure times were generated from a

discrete logistic model with covariate coefficients equal to those estimated by the Newton's method with P-spline.

Table S4 presents the variable selection performance under different interaction hierarchy structure using the simulation setting with values set from Melanoma Cancer fitted results. Simulations were designed with a strong hierarchy interaction structure (two-way interactions only for variables included as main effects). This setting shows the superior variable selection performance of our flexible algorithm under strong hierarchy conditions (Table S4). Notably, our algorithm also maintains acceptable variable selection performance even under misspecified weak or no hierarchy conditions.

3.5 Hierarchical Conditions for Time-Varying Effects

In addition the the flexibility of using different interaction hierarchy conditions, the proposed DiscBoosting procedure is capable of accommodating time-varying hierarchy conditions. Specifically, within a time-varying hierarchical framework, covariates may exhibit time-varying effects only subsequent to the selection of corresponding time-independent effects. Without this hierarchy, variables may be selected as time-varying at any point. Table S5 shows that using a time-varying hierarchy helps avoid mistakenly identifying noise as significant changes over time. This approach significantly improves how accurately we can select important variables, particularly in high dimensional settings.

References

Boyd, S., & Vandenberghe, L. (2004). *Convex Optimization.* Cambridge University Press.

Kalbfleisch, J. D., & Prentice, R. L. (2002). *The Statistical Analysis of Failure Time Data.* John Wiley & Sons.

Yan, J., & Huang, J. (2012). Model selection for Cox models with time-varying coefficients. *Biometrics*, 68(2), 419–428.

Yuan, M., & Lin, Y. (2006). Model selection and estimation in regression with grouped variables. *Journal of the Royal Statistical Society Series B: Statistical Methodology*, 68(1), 49–67.

Fig. S2: This figure shows the cumulative counts of variables selected by the DiscBoosting algorithm applied to melanoma cancer data, using the parameter re-estimation BIC stopping criterion. The x-axis represents the number of iterations of the algorithm. The y-axis represents the number of times each variable is selected (cumulative count). Solid lines indicate variables selected as time-independent, while dotted lines indicate time-varying variables. The vertical line shows where the parameter re-estimation BIC stopping criterion stopped the algorithm.

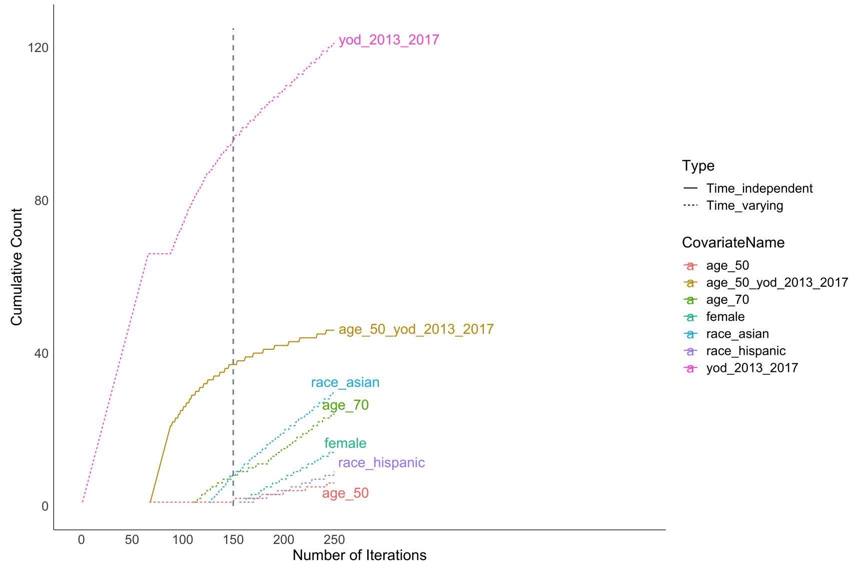

Table S2: Simulation results for the selection performance under different hierarchy structures. False positive (FP), False negative (FN), Sensitivity (SE), Specificity (SP), Positive predictive value (PPV), and Negative predictive value (NPV) are reported as mean (sd) over the simulations. In each scenario, 100 data replicates were generated with a sample size of 2,000. The model incorporates 40 main covariates. The first 20 covariates ($j = 1, \ldots, 20$) have time-varying effects generated by repeating the sequence of functions: $1+\cos(\pi t/50)$, $-1+\exp(-0.25t)$, $1-\cos(\pi t/30)$, $\sin(3\pi t/80)$, and $-\sin(\pi t/30)$. The remaining 20 main covariates ($j = 21, \ldots, 40$) have time-independent effects alternating between -1 and 1. Among the 780 possible two-way interaction terms, only six specific interactions ($z_1z_2, z_2z_4, z_5z_6, z_{10}z_{20}, z_{12}z_{25}, z_{18}z_{40}$) have non-zero effects, with coefficients equal to $(1, 1, 1, 1, 1, -1)$. We evaluated four distinct scenarios: Full model, where all covariates were treated as time-varying and all two-way interactions are included. The simulation setting was evaluated using parameter re-estimation BIC stopping criteria. The performance is evaluated across three user-specified hierarchical constraints: strong hierarchy, weak hierarchy, and no hierarchy. Notably, the FP values for the 'Overall' and 'Interactions' metrics are identical; this is because all 40 main effects were generated as true signals.

Measure	Hierarchy structure	FP (sd)	FN (sd)	SE (sd)	SP (sd)	PPV (sd)	NPV (sd)
Overall	Strong hierarchy	0.60 (0.77)	5.28 (3.31)	0.89 (0.07)	1.00 (0.00)	0.99 (0.02)	0.99 (0.00)
	Weak hierarchy	0.39 (0.63)	4.66 (3.01)	0.90 (0.07)	1.00 (0.00)	0.99 (0.01)	0.99 (0.00)
	No hierarchy	0.18 (0.41)	3.96 (2.63)	0.91 (0.06)	1.00 (0.00)	1.00 (0.01)	0.99 (0.00)
TV	Strong hierarchy	12.08 (2.33)	4.09 (2.24)	0.80 (0.11)	0.98 (0.00)	0.57 (0.05)	0.99 (0.00)
	Weak hierarchy	12.07 (2.58)	4.66 (2.51)	0.77 (0.13)	0.98 (0.00)	0.56 (0.06)	0.99 (0.00)
	No hierarchy	12.29 (2.35)	4.14 (2.61)	0.79 (0.13)	0.98 (0.00)	0.56 (0.06)	0.99 (0.00)
Interactions	Strong hierarchy	0.60 (0.77)	1.25 (0.95)	0.79 (0.16)	1.00 (0.00)	0.90 (0.12)	1.00 (0.00)
	Weak hierarchy	0.39 (0.63)	0.08 (0.27)	0.99 (0.05)	1.00 (0.00)	0.95 (0.08)	1.00 (0.00)
	No hierarchy	0.18 (0.41)	0.00 (0.00)	1.00 (0.00)	1.00 (0.00)	0.97 (0.06)	1.00 (0.00)

Table S3: Simulation results. Estimation results for covariate effects: Absolute bias, IMSE, and SD. In each scenario, 100 data replicates were generated with a sample size of 2000. The model is fitted using a fixed number of $K = 7$ basis functions. The model incorporates 40 main covariates. The first 20 covariates ($j = 1, \ldots, 20$) have time-varying effects generated by repeating the sequence of functions: $1 + \cos(\pi t/50)$, $-1 + \exp(-0.25t)$, $1 - \cos(\pi t/30)$, $\sin(3\pi t/80)$, and $-\sin(\pi t/30)$. The remaining 20 main covariates ($j = 21, \ldots, 40$) have time-independent effects alternating between -1 and 1. Among the 780 possible two-way interaction terms, only six specific interactions ($z_1z_2, z_2z_4, z_5z_6, z_{10}z_{20}, z_{12}z_{25}, z_{18}z_{40}$) have non-zero effects, with coefficients equal to $(1, 1, 1, 1, 1, -1)$. We evaluated four distinct scenarios: Full model, where all covariates were treated as time-varying and all two-way interactions are included. We evaluated three distinct scenarios: Full model, where all covariates were treated as time-varying and all two-way interactions are included; Re-estimation BIC, where DiscBoosting with parameter re-estimation BIC as the stopping criterion was used for covariate selection (evaluated under strong hierarchy, weak hierarchy, and no hierarchy constraints); and Benchmark, an idealized scenario incorporating only covariates with true signals for comparison purposes. Penalization coefficient of P-spline and Smoothing-spline was chosen by TIC. The results are expressed in terms of hundreds, with the decimal places preserved.

Scenario	Estimation	Bias	SD	IMSE
Benchmark	NR (P-spline)	2.54	1.32	1.71
	NR (Smooth-spline)	2.37	1.49	1.59
Full model	NR (P-spline)	17.23	17.32	5.98
	NR (Smooth-spline)	28.76	28.02	20.11
Re-estimation BIC (Strong hierarchy)	NR (P-spline)	2.54	1.32	1.71
	NR (Smooth-spline)	2.37	1.49	1.59
Re-estimation BIC (Weak hierarchy)	NR (P-spline)	2.16	1.28	1.29
	NR (Smooth-spline)	2.00	1.42	1.20
Re-estimation BIC (No hierarchy)	NR (P-spline)	1.96	1.19	1.09
	NR (Smooth-spline)	1.80	1.32	0.99

Table S4: Simulation results. Evaluation of variable selection performance in a setting that mimics the melanoma survival data. False positive (FP) was the number of predictors selected by the algorithms, while the true effect was zero. True positive (TP) was the number of predictors correctly identified. False negative (FN) was the number of true signals that the algorithms did not select. True negative (TN) was the number of noises correctly identified as having no effect. Sensitivity (SE) was calculated using the number of correctly chosen signals divided by the number of true signals. Specificity (SP) was calculated using the number of correctly ignored noises divided by the number of noises. Positive predictive value (PPV) was calculated using TP divided by the number of selected predictors. Negative predictive value (NPV) was calculated using TN divided by the number of ignored predictors. The model is fitted with a fixed number of $K = 7$ basis functions. The covariates are simulated based on Melanoma cancer data fitted results. The following interaction term $z_1 z_8$ has non-zero effects equal to -0.27. The 'TI or TV' metric assesses the algorithms' ability to select either time-independent or time-varying effects or both accurately, whereas 'TV' focuses on the selection of time-varying effects. The simulation setting was evaluated using parameter re-estimation BIC stopping criteria. The performance is evaluated across three user-specified hierarchical constraints: strong hierarchy, weak hierarchy, and no hierarchy.

Stopping criteria	Measure	Hierarchy	FP (sd)	FN (sd)	SE (sd)	SP (sd)	PPV (sd)	NPV (sd)
Parameter re-estimation BIC	TI or TV	Strong hierarchy	0.14 (0.51)	1.22 (0.61)	0.76 (0.12)	1.00 (0.02)	0.98 (0.09)	0.04 (0.02)
		Weak hierarchy	0.92 (0.27)	1.13 (0.56)	0.77 (0.11)	0.97 (0.01)	0.81 (0.06)	0.03 (0.02)
		No hierarchy	0.93 (0.29)	1.13 (0.56)	0.77 (0.11)	0.97 (0.01)	0.81 (0.06)	0.03 (0.02)
	TV	Strong hierarchy	0.03 (0.17)	1.98 (0.71)	0.50 (0.18)	0.99 (0.04)	0.99 (0.06)	0.32 (0.09)
		Weak hierarchy	0.00 (0.00)	1.95 (0.74)	0.51 (0.19)	1.00 (0.00)	1.00 (0.00)	0.32 (0.10)
		No hierarchy	0.00 (0.00)	1.95 (0.74)	0.51 (0.19)	1.00 (0.00)	1.00 (0.00)	0.32 (0.10)
	Interactions	Strong hierarchy	0.07 (0.26)	0.34 (0.48)	0.66 (0.48)	1.00 (0.01)	0.96 (0.13)	0.01 (0.02)
		Weak hierarchy	0.92 (0.27)	0.27 (0.45)	0.73 (0.45)	0.97 (0.01)	0.42 (0.28)	0.01 (0.02)
		No hierarchy	0.93 (0.29)	0.27 (0.45)	0.73 (0.45)	0.97 (0.01)	0.41 (0.28)	0.01 (0.02)

Table S5: Simulation results. Evaluation of variable selection performance under time-varying hierarchy conditions. False positive (FP) was the number of predictors selected by the algorithms, while the true effect was zero. True positive (TP) was the number of predictors correctly identified. False negative (FN) was the number of true signals that the algorithms did not select. True negative (TN) was the number of noises correctly identified as having no effect. Sensitivity (SE) was calculated using the number of correctly chosen signals divided by the number of true signals. Specificity (SP) was calculated using the number of correctly ignored noises divided by the number of noises. Positive predictive value (PPV) was calculated using TP divided by the number of selected predictors. Negative predictive value (NPV) was calculated using TN divided by the number of ignored predictors. The simulations involved a sample size of 500 patients with the number of covariates set at 100 and 1,000. Each simulation scenario was replicated 100 times. The covariate vector's non-zero components were defined as follows: $\beta_1(t) = 1$, $\beta_2(t) = \cos(\pi t/50)$, $\beta_3(t) = -1$, $\beta_4(t) = \sin(3\pi t/80)$, $\beta_5(t) = -1 + \exp(-0.25t)$. Both continuous and binary covariates were included in the analysis. The 'TI or TV' metric assesses the algorithms' ability to select either time-invariant or time-varying effects accurately. 'TI' focuses on the selection of time-independent effects, and 'TV' focuses on the selection of time-varying effects. Parameter re-estimation BIC stopping criteria is employed. Under time-varying hierarchy, covariates may exhibit time-varying effects only after the corresponding time-independent effects have been selected. Without this hierarchy, variables may be selected as time-varying at any point. This study contrasts the selection performance under scenarios with and without the time-varying hierarchy.

Number of covariates	Variable	Measure	Method	FP (sd)	FN (sd)	SE (sd)	SP (sd)	PPV (sd)	NPV (sd)
P = 100	Continuous	TI or TV	Without time-varying hierarchy	0.24 (0.61)	0.80 (0.61)	0.84 (0.12)	1.00 (0.01)	0.96 (0.09)	0.99 (0.01)
		$\beta_{1,2,3,4,5}(t) \neq 0$	With time-varying hierarchy	0.00 (0.00)	1.18 (0.39)	0.76 (0.08)	1.00 (0.00)	1.00 (0.00)	0.99 (0.00)
		TI	Without time-varying hierarchy	1.03 (0.56)	0.28 (0.45)	0.86 (0.23)	0.99 (0.01)	0.65 (0.17)	1.00 (0.00)
		$\beta_{1,3}(t) \neq 0$	With time-varying hierarchy	1.82 (0.39)	0.00 (0.00)	1.00 (0.00)	0.98 (0.00)	0.53 (0.06)	1.00 (0.00)
		TV	Without time-varying hierarchy	1.36 (1.06)	1.33 (0.85)	0.56 (0.28)	0.99 (0.01)	0.60 (0.27)	0.99 (0.01)
		$\beta_{2,4,5}(t) \neq 0$	With time-varying hierarchy	1.06 (0.75)	1.77 (0.69)	0.41 (0.23)	0.99 (0.01)	0.58 (0.30)	0.98 (0.01)
	Binary	TI or TV	Without time-varying hierarchy	0.72 (0.88)	1.67 (0.89)	0.67 (0.18)	0.99 (0.01)	0.86 (0.16)	0.98 (0.01)
		$\beta_{1,2,3,4,5}(t) \neq 0$	With time-varying hierarchy	0.07 (0.26)	1.70 (0.61)	0.66 (0.12)	1.00 (0.00)	0.98 (0.06)	0.98 (0.01)
		TI	Without time-varying hierarchy	0.35 (0.52)	1.41 (0.61)	0.30 (0.30)	1.00 (0.01)	0.76 (0.37)	0.99 (0.01)
		$\beta_{1,3}(t) \neq 0$	With time-varying hierarchy	1.49 (0.58)	0.12 (0.36)	0.94 (0.18)	0.98 (0.01)	0.57 (0.13)	1.00 (0.00)
		TV	Without time-varying hierarchy	2.31 (1.05)	1.52 (0.76)	0.49 (0.25)	0.98 (0.01)	0.39 (0.16)	0.98 (0.01)
		$\beta_{2,4,5}(t) \neq 0$	With time-varying hierarchy	1.67 (0.57)	1.75 (0.64)	0.42 (0.21)	0.98 (0.01)	0.42 (0.20)	0.98 (0.01)
P = 1000	Continuous	TI or TV	Without time-varying hierarchy	0.58 (1.10)	1.02 (0.57)	0.80 (0.11)	1.00 (0.00)	0.91 (0.15)	1.00 (0.00)
		$\beta_{1,2,3,4,5}(t) \neq 0$	With time-varying hierarchy	0.00 (0.00)	1.21 (0.46)	0.76 (0.09)	1.00 (0.00)	1.00 (0.00)	1.00 (0.00)
		TI	Without time-varying hierarchy	0.97 (0.52)	0.34 (0.48)	0.83 (0.24)	1.00 (0.00)	0.65 (0.17)	1.00 (0.00)
		$\beta_{1,3}(t) \neq 0$	With time-varying hierarchy	1.79 (0.46)	0.00 (0.00)	1.00 (0.00)	1.00 (0.00)	0.54 (0.07)	1.00 (0.00)
		TV	Without time-varying hierarchy	1.74 (1.42)	1.47 (0.83)	0.51 (0.28)	1.00 (0.00)	0.52 (0.27)	1.00 (0.00)
		$\beta_{2,4,5}(t) \neq 0$	With time-varying hierarchy	1.13 (0.72)	1.73 (0.74)	0.42 (0.25)	1.00 (0.00)	0.54 (0.31)	1.00 (0.00)
	Binary	TI or TV	Without time-varying hierarchy	3.02 (2.44)	2.52 (0.90)	0.50 (0.18)	1.00 (0.00)	0.52 (0.23)	1.00 (0.00)
		$\beta_{1,2,3,4,5}(t) \neq 0$	With time-varying hierarchy	0.35 (0.64)	1.74 (0.65)	0.65 (0.13)	1.00 (0.00)	0.92 (0.14)	1.00 (0.00)
		TI	Without time-varying hierarchy	0.28 (0.47)	1.51 (0.54)	0.24 (0.27)	1.00 (0.00)	0.80 (0.36)	1.00 (0.00)
		$\beta_{1,3}(t) \neq 0$	With time-varying hierarchy	1.79 (0.84)	0.18 (0.41)	0.91 (0.21)	1.00 (0.00)	0.52 (0.17)	1.00 (0.00)
		TV	Without time-varying hierarchy	4.27 (2.55)	2.08 (0.76)	0.31 (0.25)	1.00 (0.00)	0.19 (0.16)	1.00 (0.00)
		$\beta_{2,4,5}(t) \neq 0$	With time-varying hierarchy	1.84 (0.77)	1.77 (0.76)	0.41 (0.25)	1.00 (0.00)	0.39 (0.23)	1.00 (0.00)

Table S6: Simulation results. Estimation results for covariate effects: Absolute bias, IMSE, SD, and average negative log likelihood divided by the sample size on the training and testing data sets. In each scenario, 100 data replicates were generated with a sample size of 1,000 and 4,000. The model is fitted using a fixed number of $K = 7$ basis functions. The covariates with time-varying effects are $\beta_j(t) = (1 + \cos(\pi t/50), -1 + \exp(-0.25t), 1 - \cos(\pi t/30), \sin(3\pi t/80), -\sin(\pi t/30)), j = 1, \ldots, 5$. The remain 10 main covariates have time-independent effects with $\beta_j(t) = (-1, -1, -1, 1, 1, 1, 1, 0, 0, 0), j = 6, \ldots, 15$. The following interaction terms $z_1z_2, z_1z_3, z_1z_9, z_5z_{10}, z_6z_7$ have non-zero effects with coefficients equal to $(1, 1, 1, -1, -1)$. We evaluated four distinct scenarios: Full model, where all covariates were treated as time-varying and all two-way interactions are included; Boosting BIC, with covariates selected via DiscBoosting using the Boosting BIC stopping criterion; Re-estimation BIC, where DiscBoosting with parameter re-estimation BIC as the stopping criterion was used for covariate selection; and Benchmark, an idealized scenario incorporating only covariates with true signals for comparison purposes. No Re-estimation refers to boosting estimation. Penalization coefficient of P-spline and Smoothing-spline was chosen by TIC. The results are expressed in terms of hundreds, with the decimal places preserved.

Sample Size	Scenario	Estimation	Bias	SD	IMSE	$-\ell$ - training	$-\ell$ - testing
n = 1000	Benchmark	NR (no penalty)	3.07	3.95	1.13	146.53	156.35
		NR (P-spline)	2.68	3.34	0.63	147.62	150.91
		NR (Smooth-spline)	2.80	3.52	0.75	147.30	151.52
	Full model	NR (no penalty)	28.73	35.66	13.33	137.34	180.78
		NR (P-spline)	24.58	29.93	7.78	141.58	163.70
		NR (Smooth-spline)	26.22	32.09	9.33	140.39	167.78
	Boosting BIC	NR (no penalty)	9.66	12.68	5.01	145.68	169.38
		NR (P-spline)	8.76	10.54	3.66	148.10	157.78
		NR (Smooth-spline)	9.04	11.40	4.02	147.70	158.77
		No Re-estimation	14.15	4.85	8.49	179.04	182.61
	Re-estimation BIC	NR (P-spline)	8.26	8.77	3.60	149.49	156.41
		NR (Smooth-spline)	8.41	9.03	3.71	149.47	156.64
n = 4000	Benchmark	NR (no penalty)	1.46	1.80	0.21	149.10	151.90
		NR (P-spline)	1.59	1.93	0.26	149.30	150.46
		NR (Smooth-spline)	1.52	1.64	0.20	149.67	150.26
	Full model	NR (no penalty)	11.17	14.00	1.72	147.02	157.73
		NR (P-spline)	10.78	12.38	1.48	148.37	152.11
		NR (Smooth-spline)	10.79	12.40	1.49	148.38	152.12
	Boosting BIC	NR (no penalty)	4.91	8.00	0.82	147.96	156.06
		NR (P-spline)	5.06	7.82	0.87	148.80	151.73
		NR (Smooth-spline)	4.89	7.80	0.83	148.55	151.71
		No Re-estimation	10.85	4.47	4.18	178.24	177.35
	Re-estimation BIC	NR (P-spline)	4.05	5.44	0.72	149.40	151.00
		NR (Smooth-spline)	4.00	6.11	0.74	148.87	151.63
